# Reproducibility of clinical research in critical care: a scoping review

**DOI:** 10.1186/s12916-018-1018-6

**Published:** 2018-02-21

**Authors:** Daniel J. Niven, T. Jared McCormick, Sharon E. Straus, Brenda R. Hemmelgarn, Lianne Jeffs, Tavish R. M. Barnes, Henry T. Stelfox

**Affiliations:** 1 Department of Critical Care Medicine, University of Calgary, 3134 Hospital Drive NW, Calgary, AB T2N 2T9 Canada; 2 Department of Anesthesiology and Pain Medicine, University of Ottawa, 1053 Carling Avenue, B302, Ottawa, ON K1Y 4E9 Canada; 3 Li Ka Shing Knowledge Institute of St. Michael’s Hospital, University of Toronto, 30 Bond Street, Toronto, ON M5B 1W8 Canada; 4 Department of Community Health Sciences, University of Calgary, 3280 Hospital Drive NW, Calgary, AB T2N 4Z6 Canada; 5 St. Michael’s Hospital Volunteer Association Chair in Nursing and Scientist with the Keenan Research Center, Li Ka Shing Knowledge Institute of St. Michael’s Hospital, Institute of Health Policy Management and Faculty of Nursing, University of Toronto, 30 Bond Street, Toronto, ON M5B 1W8 Canada

**Keywords:** Reproducibility, Replication research, Adoption, De-adoption, ICU, Critical care, Intensive care

## Abstract

**Background:**

The ability to reproduce experiments is a defining principle of science. Reproducibility of clinical research has received relatively little scientific attention. However, it is important as it may inform clinical practice, research agendas, and the design of future studies.

**Methods:**

We used scoping review methods to examine reproducibility within a cohort of randomized trials examining clinical critical care research and published in the top general medical and critical care journals. To identify relevant clinical practices, we searched the *New England Journal of Medicine, The Lancet,* and *JAMA* for randomized trials published up to April 2016. To identify a comprehensive set of studies for these practices, included articles informed secondary searches within other high-impact medical and specialty journals. We included late-phase randomized controlled trials examining therapeutic clinical practices in adults admitted to general medical-surgical or specialty intensive care units (ICUs). Included articles were classified using a reproducibility framework. An original study was the first to evaluate a clinical practice. A reproduction attempt re-evaluated that practice in a new set of participants.

**Results:**

Overall, 158 practices were examined in 275 included articles. A reproduction attempt was identified for 66 practices (42%, 95% CI 33–50%). Original studies reported larger effects than reproduction attempts (primary endpoint, risk difference 16.0%, 95% CI 11.6–20.5% vs. 8.4%, 95% CI 6.0–10.8%, *P* = 0.003). More than half of clinical practices with a reproduction attempt demonstrated effects that were inconsistent with the original study (56%, 95% CI 42–68%), among which a large number were reported to be efficacious in the original study and to lack efficacy in the reproduction attempt (34%, 95% CI 19–52%). Two practices reported to be efficacious in the original study were found to be harmful in the reproduction attempt.

**Conclusions:**

A minority of critical care practices with research published in high-profile journals were evaluated for reproducibility; less than half had reproducible effects.

**Electronic supplementary material:**

The online version of this article (10.1186/s12916-018-1018-6) contains supplementary material, which is available to authorized users.

## Background

Owing to harms associated with early acceptance of scientific claims that are subsequently not reproducible [1], the reproducibility of science has garnered attention from high-profile journals [2–6] and mainstream media [7–9]. Most research pertaining to scientific reproducibility concentrates within biomedical sciences, and suggests that 10–25% of the findings from biomedical research are reproducible [5, 6, 10]. Reproducibility within clinical research has received relatively less scientific attention, despite being equally important as it may inform clinical practice, research agendas, and the design of future studies.

In biomedical research, it is common to evaluate an experiment’s ‘*methodological reproducibility’* through repeating previously performed experiments using exactly the same methods, data, and tools as the original experiment [11]. Assessing methodological reproducibility requires accurate reporting of methods in the original study, and an experimental population that can be easily accessed or recreated. Clinical research is typically evaluated for results or inferential reproducibility, wherein ‘*results reproducibility’* refers to corroborating the results of an original study by repeating the original methods in a new set of participants and ‘*inferential reproducibility*’ refers to the ability of independent analyses to draw the same conclusions from a given dataset [11]. Clinical studies examining results reproducibility of an original study may be further described as a retest (direct) or an approximate (conceptual) reproduction attempt [12, 13]. A retest reproduction attempt repeats exactly the methodology of the original study in another group of participants, whereas an approximate reproduction attempt may deviate slightly from the methodology employed in the original study [12, 13].

Most studies that have examined reproducibility within clinical research assessed results reproducibility. Estimates from these studies suggest that less than half of reproduction attempts report results that are consistent with the original study [14–18]. However, most of these studies did not employ systematic review methodology, and/or employed definitions of reproducibility that are difficult to reliably operationalize [14–18]. We used scoping review methodology to systematically examine results reproducibility (inclusive of both retest and approximate subtypes) of clinical research. Scoping reviews are a type of knowledge synthesis designed to provide a broad perspective of the literature, set research agendas and provide high-level information for decision-makers [19–21], and represent an ideal means of systematically studying reproducibility. Similar to a recent study examining reproducibility in psychological science [4], for reasons of feasibility, we focused our study on one test clinical discipline, namely adult critical care medicine.

## Methods

### Research approach

We used two phases of electronic database searching to identify the target cohort of articles. To identify clinical practices relevant to a broad audience of critical care providers [22], and which were the subject of potentially high-profile research [23], our primary search involved randomized controlled trials (RCTs) examining the efficacy, effectiveness, or safety of therapeutic clinical practices among adults admitted to intensive care units (ICUs) published in the three medical journals with the highest impact factors, namely the *New England Journal of Medicine, The Lancet,* and *JAMA*. To identify a comprehensive set of studies for the clinical practices identified in the primary search, we conducted a secondary search for articles examining these practices published in other high-profile general medical or critical care specialty journals (*Annals of Internal Medicine*, *BMJ*, *American Journal of Respiratory and Critical Care Medicine*, *Chest*, *Critical Care Medicine*, *Intensive Care Medicine*, and *Critical Care*) [24]. Results from the two sets of searches established the target ‘cohort’ of articles that was subsequently analyzed within a framework to describe reproducibility of experimental clinical research (Table 1). Our methods are outlined in a detailed, published protocol [25] and depicted within Additional file 1: Figure S1. The published protocol indicates intention to include systematic reviews, systematic reviews with meta-analyses, and studies examining the clinical effects of diagnostic interventions within the target cohort of articles; however, at the request of the reviewers, these studies were removed from this manuscript.

**Table 1:** Reproducibility framework, terms, and definitions

Reproducibility component	Definition
Unique clinical practice	A specific intervention applied to patients with a specific target condition (e.g., therapeutic hypothermia for patients with traumatic brain injury)
Reported effect of clinical practice	
Efficacy	For the primary outcome, statistically significant increased risk of a positive outcome, or decreased risk of a negative outcome
Harm	For the primary outcome or any pre-specified secondary or safety outcome, statistically significant increased risk of a negative outcome, or decreased risk of a positive outcome^a^
Lack of efficacy	For the primary outcome, a non-statistically significant change
Type of results reproducibility [12]	
Re-test reproduction attempt	For a given clinical practice, a study that re-examined the results of an original study in another group of participants using methodology identical to that of the original study^b^
Approximate reproduction attempt	For a given clinical practice, a study that re-examined the results of an original study in another group of participants using methodology with minor changes to the population, setting, treatment, outcomes, and/or analyses relative to the original study^b^
Reproducibility classification	
Original study	First randomized controlled trial to examine the effects of a clinical practice^c^
Reproduction attempt	Re-test or approximate reproduction attempt for an original study
Consistent effect estimate between original study and reproduction attempt	Clinical practice effect reported in the reproduction attempt was congruent with that in the original study: - Efficacy/efficacy - Lack of efficacy/lack of efficacy - Harm/harm
Inconsistent effect estimate between original study and reproduction attempt	Clinical practice effect reported in the reproduction attempt was different from that in the original study: - Efficacy/harm - Efficacy/lack of efficacy - Harm/lack of efficacy - Harm/efficacy - Lack of efficacy/harm - Lack of efficacy/efficacy

^a^Where there was a significant positive effect for the primary outcome, and a significant negative effect for a safety outcome, practice classification was based on the relative importance of each outcome. For example, if survival was improved, but there was an increased incidence of adverse drug reaction, the practice was classified as having efficacy

^b^Sample size of reproduction attempt was required to be at least 90% that of the original study [14]

^c^Early phase trials did not count as an original study; these were defined as those for which the main objective was to evaluate the feasibility of processes (recruitment, randomization, blinding, outcome assessment, etc.) required to examine the effect of the clinical practice in a later phase clinical trial [53]

### Eligibility criteria

For the primary search, studies were retained if (1) study design was a late-phase RCT, (2) the study population included adults (mean age ≥ 18 years) admitted to general medical-surgical or specialty ICUs [26], and (3) the effect of a therapeutic clinical practice was reported. Late-phase RCTs were phase III or IV studies that examined the efficacy, effectiveness, or safety of a given therapy [27]. Studies were excluded if (1) study participants were primarily admitted to coronary care units [28], (2) the clinical practice was provided exclusively in the pre-hospital setting, or (3) the study examined diagnostic accuracy or outcomes associated with the use of a diagnostic intervention. For the secondary searches, studies were retained if they fit the primary search eligibility criteria AND represented an ‘*original study’* OR a ‘*reproduction attempt*’ of a study identified in the primary search (Table 1) [25].

### Search strategy and data sources

For the primary search, we used MEDLINE, the Cochrane Central Register of Controlled Trials, and the American College of Physicians (ACP) Journal Club to search for relevant articles published in the three highest-impact medical journals from database inception (1946) to April 4, 2016. The MEDLINE search (available in Additional file 1: Online Appendix) was peer-reviewed by an experienced librarian using the Peer Review of Electronic Search Strategies (PRESS) checklist [29].

For secondary searches, the PubMed ‘related articles’ feature was used to conduct targeted searches for articles related to those included from the primary search, published in the other aforementioned general medical and critical care journals (Additional file 1: Figure S1). Additional sources of articles included bibliographies of included articles, and international clinical trial registries [30, 31].

### Study selection

A screening form was independently calibrated by three team members with a random sample of 50 articles. Once consistent selection was achieved (κ ≥ 0.8) [32], a two-stage process was used to independently and in duplicate screen all articles identified by the searches. First, titles and abstracts were reviewed to determine whether the studies met inclusion or exclusion criteria. Second, the full text of any study classified as ‘*include’* or ‘*unclear’* after title and abstract review was assessed to determine whether it met inclusion criteria. Eligibility disagreements were resolved by consensus or arbitration by an additional reviewer. Agreement was quantified with the κ statistic [32].

### Data extraction and analysis

Data was extracted independently and in duplicate using a predesigned electronic form, which was pilot tested with a random sample of 10 articles. Once data was consistently abstracted (κ ≥ 0.8) [32], reviewers proceeded with data extraction for the full set of included articles. Extracted data were related to the study itself, the study participants, the practice under investigation, and the primary outcome.

Included articles were analyzed using a framework to describe reproducibility of experimental clinical research (Table 1). The framework was developed using approaches outlined in previous research [4, 12, 14–16]. First, included articles were categorized according to the unique clinical practice they examined (e.g., therapeutic hypothermia for anoxic brain injury). Second, data for a study’s primary outcome and any secondary safety outcomes were used to classify the effect of each unique practice reported in each article as efficacy, lack of efficacy, or harm [33]. Where there was a significant positive effect reported for the primary outcome, and a significant negative effect reported for a safety outcome, practice classification was based on the relative importance of each outcome. For example, if survival was improved, but there was an increased incidence of adverse drug reaction, the practice was classified as having efficacy. Third, within each unique clinical practice, relevant articles were classified as an ‘*original study’* or a ‘*reproduction attempt*’. An original study was chronologically the first experimental study to examine the effects of a clinical practice. A reproduction attempt was any subsequent article that (intentionally or unintentionally) endeavored to re-examine the results of the original by repeating the methodology in another group of participants. To be considered a reproduction attempt the sample size had to be at least 90% that of the original RCT [14]. Finally, using the effect reported for each practice, original studies and reproduction attempts were further classified according to whether they demonstrated ‘*consistent effect estimates’* (e.g., efficacy in original study and reproduction attempt) or ‘*inconsistent effect estimates*’ (e.g., efficacy in original study and lack of efficacy in reproduction attempt). Practices with ‘*consistent effect estimates*’ denoted those with reproducible results, whereas practices with ‘*inconsistent effect estimates*’ denoted those with non-reproducible results.

Normally distributed data were reported as mean and 95% confidence interval (CI). Skewed data were transformed using logarithms and reported as geometric mean and 95% CI. Nominal data were summarized using counts with percentages, or percentages with 95% CI where appropriate. Statistical comparisons between original studies and reproduction attempts were performed using mixed effects logistic regression with clustering at the level of the individual clinical practice. For all other comparisons, Fisher’s exact test, χ^2^, or Student’s *t* test were used, as appropriate. All analyses were conducted using Stata version 14.2 (Stata Corp, College Station, TX, USA) and statistical significance was set at *P* < 0.05.

## Results

**Fig. 1:**
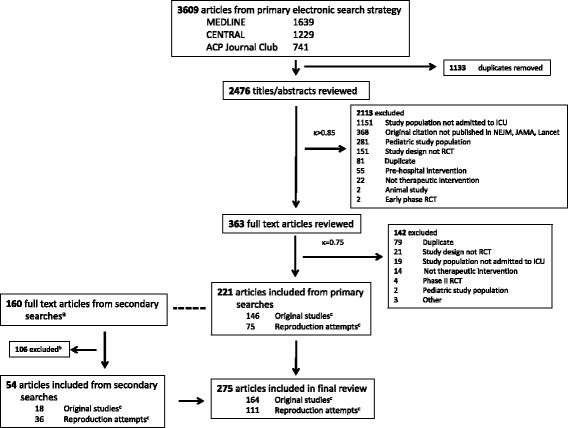
Details of the study selection process. Detailed legend: ^a^Studies included from primary search informed the secondary searches (dashed line). Studies identified in the secondary searches that were published before the corresponding study in the primary search were classified as the original study for that practice, whereas those published after the corresponding study in the primary search were classified as a reproduction attempt. ^b^Studies were excluded if they did not meet eligibility criteria or did not represent an original study or reproduction attempt for any study that was included from the primary search. ^c^Classification as original study or reproduction attempt determined after analyzing final cohort of articles in context of reproducibility framework (Table 1)

From 2636 unique articles, 275 relevant articles were identified that reported on 158 unique clinical practices in 283 studies (Fig. 1). Because one article could report on the effects of more than one practice (e.g., factorial RCT), we used the term ‘study’ to refer to any comparison of an intervention to a control. Accordingly, there were more studies than articles because seven factorial RCTs reported results for two clinical practices in the same article [34–40], and one article reported on the results of two separate RCTs [41]. Most included studies were published after 1990 (*n* = 259, 92%), and examined the effects of drugs (*n* = 134, 47%) or devices (*n* = 95, 34%) in patients with respiratory failure (*n* = 102, 36%). Characteristics of the included studies are described in Table 2, and bibliographic details appear in Additional file 1: Tables S1–S5.

**Table 2:** Characteristics of included studies classified according to reproduction attempts

	Practices WITHOUT a reproduction attempt (*n* = 92)	Practices with CONSISTENT EFFECT between original study and reproduction attempt (*n* = 28)		Practices with INCONSISTENT EFFECT between original study and reproduction attempt (*n* = 35)	
Characteristic, n (%)^a^	Original study (*n* = 93)^b^	Original study (*n* = 30)^b^	Reproduction attempt (*n* = 39)^b^	Original study (*n* = 37)^b^	Reproduction attempt (*n* = 63)^b^
Primary electronic search	93 (100)	20 (67)	25 (64)	29 (78)	51 (81)
Secondary electronic search	0 (0)	10 (33)	14 (36)	8 (22)	12 (19)
Continent of origin					
North America	44 (47)	13 (43)	12 (31)	18 (49)	20 (32)
Europe	42 (45)	15 (50)	21 (54)	15 (41)	37 (59)
Australasia	7 (8)	1 (3)	4 (10)	2 (5)	4 (6)
Other	0 (0)	1 (3)	2 (5)	2 (5)	2 (3)
Year of publication^e,f^					
Before 1980	3 (3)	0 (0)	0 (0)	1 (3)	0 (0)
1980–1989	7 (8)	2 (7)	1 (3)	4 (11)	3 (5)
1990–1999	25 (27)	14 (47)	6 (15)	13 (35)	12 (19)
2000–2009	20 (22)	11 (37)	21 (54)	17 (46)	21 (33)
2010 or later	38 (41)	3 (10)	11 (28)	2 (5)	27 (43)
Participating center type^f^					
University affiliated	38 (41)	17 (57)	19 (49)	29 (78)	23 (37)
Mixed university affiliated and non-affiliated	11 (12)	5 (17)	11 (28)	1 (3)	13 (21)
Unclear	44 (47)	8 (27)	9 (23)	7 (19)	27 (43)
No. of centres, mean (95% CI)^e,f^	7.2 (5.1–9.9)	4.0 (2.2–7.0)	9.1 (5.4–15.3)	2.9 (1.8–4.6)	16.2 (11.1–23.4)
1^e,f^	25 (26)	11 (37)	9 (23)	17 (46)	7 (11)
2–4^e,f^	13 (14)	8 (27)	4 (10)	8 (22)	5 (8)
5–9^e,f^	12 (13)	3 (10)	5 (13)	6 (16)	5 (8)
≥ 10^e,f^	44 (47)	8 (27)	21 (54)	6 (16)	46 (73)
No. of participants, mean (95% CI)^e,f^	362.6 (266.3–493.6)	155.9 (105.5–230.4)	344.8 (223.9–531.2)	146.7 (96.5–222.8)	548.5 (408.8–735.7)
< 100^d,e,f,g^	17 (18)	9 (30)	8 (21)	16 (43)	3 (5)
100–499^d,e,f,g^	40 (43)	15 (50)	12 (31)	16 (43)	29 (46)
500–999^d,e,f,g^	16 (17)	6 (20)	13 (33)	1 (3)	13 (21)
≥ 1000^d,e,f,g^	20 (22)	0 (0)	6 (15)	4 (11)	18 (29)
Target condition					
General critical illness	10 (11)	2 (7)	2 (5)	5 (14)	12 (19)
Respiratory	24 (26)	13 (43)	19 (49)	13 (35)	23 (37)
ARDS	4 (4)	5 (17)	8 (21)	5 (14)	7 (11)
Mechanical ventilation (excluding ARDS)	11 (12)	3 (10)	4 (10)	4 (11)	8 (13)
Respiratory failure (without ventilation)	9 (10)	5 (17)	7 (18)	4 (11)	8 (13)
Sepsis	13 (14)	6 (20)	7 (18)	7 (19)	14 (22)
Nosocomial complications	11 (12)	5 (17)	3 (8)	3 (8)	3 (5)
Neurological	12 (13)	2 (7)	1 (3)	5 (14)	8 (13)
Acute kidney injury	6 (6)	1 (3)	5 (13)	3 (8)	3 (5)
General resuscitation	9 (10)	0 (0)	1 (3)	1 (3)	0 (0)
Trauma	3 (3)	0 (0)	0 (0)	0 (0)	0 (0)
Other	5 (5)	1 (3)	1 (3)	0 (0)	0 (0)
Type of intervention					
Drug	48 (52)	14 (47)	16 (41)	18 (49)	26 (41)
Device/procedure	20 (22)	13 (43)	20 (51)	14 (38)	23 (37)
Protocol	11 (12)	2 (7)	2 (5)	4 (11)	13 (21)
Other	14 (15)	1 (3)	1 (3)	1 (3)	1 (1)
Intervention effect estimate^f^					
Lack of efficacy	51 (55)	16 (53)	20 (51)	10 (27)	38 (60)
Efficacy	31 (33)	11 (37)	15 (38)	23 (62)	16 (25)
Harm	11 (12)	3 (10)	4 (10)	4 (11)	9 (14)
Funding					
Non-commercial	46 (49)	12 (40)	23 (59)	9 (24)	29 (46)
Commercial	17 (18)	5 (17)	4 (10)	11 (30)	15 (24)
Both commercial and non-commercial	14 (15)	2 (7)	4 (10)	7 (19)	8 (13)
Not reported	15 (16)	10 (33)	8 (21)	10 (27)	11 (17)
None	1 (1)	1 (3)	0 (0)	0 (0)	0 (0)
Study stopped early					
Futility	2 (2)	0 (0)	3 (8)	0 (0)	5 (8)
Benefit	1 (1)	0 (0)	2 (5)	4 (11)	2 (3)
Harm	2 (2)	1 (3)	1 (3)	1 (3)	4 (6)
Recruitment/lack of funding	1 (1)	1 (3)	0 (0)	0 (0)	3 (5)

*ARDS* acute respiratory distress syndrome, *ICU* intensive care unit, *IQR* interquartile range, *RCT* randomized controlled trial

^a^Continuous data are reported as geometric mean (95% confidence interval) and nominal data as number (%)

^b^The 275 included articles described 158 unique practices that were examined in 283 studies. A ‘study’ is a comparison of an intervention with control. The number of studies exceeds the number of included articles because of 8 articles that simultaneously reported 2 separate studies [34–41]; 21 studies were excluded from the data in this table since the reproduction attempt was not yet completed for 13 studies and due to the following 8 practices for which representative studies did not consistently meet our criteria for results reproducibility: chlorhexidine skin antiseptic for central venous catheter insertion, naloxone for patients with sepsis, stress ulcer prophylaxis for prevention of gastrointestinal bleeding, systemic steroids in ARDS, pulmonary surfactant in ARDS, reduction of ventilator-associated pneumonia by various methods, trophic enteral nutrition, and daily interruption of sedatives. Data refer to 262 studies unless otherwise stated

^c^Primary electronic search: *New England Journal of Medicine, The Lancet, JAMA.* Secondary electronic search: *Annals of Internal Medicine, BMJ, American Journal of Respiratory and Critical Care Medicine, Chest, Critical Care Medicine, Intensive Care Medicine, Critical Care*, clinicaltrials.gov, controlled-trials.com, bibliographies of included studies

^d^*P* < 0.05 for comparison of reproduction attempts between practices with consistent and inconsistent effect estimates

^e^*P* < 0.05 for comparison between original evaluation and reproduction attempt among practices demonstrating consistent effects

^f^*P* < 0.05 for comparison between original evaluation and reproduction attempt among practices demonstrating inconsistent effects

^g^*P* < 0.05 for comparison of original evaluations between practices with consistent and inconsistent effect estimates

### Clinical practices without a reproduction attempt

**Fig. 2:**
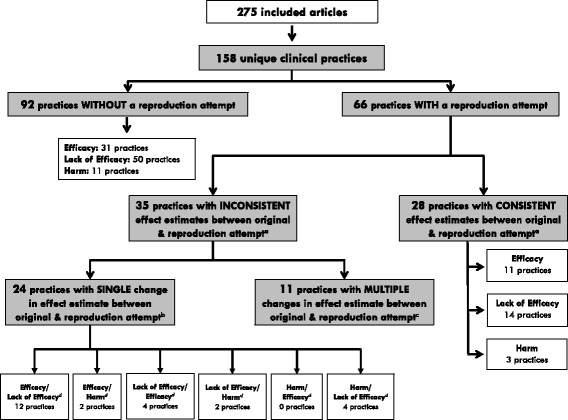
Classification of included articles and clinical practices according to the assessment of reproducibility. Detailed Legend: ^a^The sum of clinical practices with consistent (*n* = 28) and inconsistent (*n* = 35) effect estimates between original and reproduction attempts does not sum to 66 due to three practices that could not be categorized as their single reproduction attempt was in progress [38, 42, 43]. ^b^Practices wherein all reproduction attempts demonstrated similar effect estimates (e.g., all lack of efficacy). ^c^Practices wherein effect estimates from each reproduction attempt differed from the previous attempt. ^d^Each box represents the way in which the reproduction attempt changed the results of the original study (e.g., efficacy/harm represents practices wherein the original study demonstrated efficacy but reproduction attempt demonstrated harm)

Agreement for classification within our reproducibility framework was excellent (κ = 0.9). For 92 practices (58%, 95% CI 50–66%) a reproduction attempt could not be found (Fig. 2). Of these 92 practices, 31 (34%, 95% CI 24–44%) were reported to be efficacious, 50 (54%, 95% CI 43–65%) reported lack of efficacy, and 11 (12%, 95% CI 6–20%) reported harm. Practices with studies that reported efficacy commonly targeted patients with respiratory failure (n = 10, 29%), practices with studies that reported lack of efficacy commonly targeted patients with sepsis (*n* = 12, 22%), and harmful practices commonly targeted patients with neurological conditions (*n* = 3, 27%) (Additional file 1: Table S1).

### Clinical practices with a reproduction attempt

In total, 66 clinical practices (42%, 95% CI 33–50%) had one or more reproduction attempts identified. The geometric mean time from publication of the original study to publication of the first reproduction attempt was 4.6 (95% CI 3.7–5.7) years (Additional file 1: Figure S2). Original studies reported a larger effect estimate for the primary endpoint than the corresponding reproduction attempt (mean absolute risk difference 16.0%, 95% CI 11.6–20.5% vs. 8.4%, 95% CI 6.0–10.8%, *P* = 0.003). Twenty-seven of the 66 practices had at least two reproduction attempts (41%, 95% CI 28–54%). All reproduction attempts were an approximate reproduction of the corresponding original study. For three practices, the reproduction attempt was in progress [38, 42, 43]. Of the remaining 63 practices, the original study and reproduction attempt demonstrated consistent effect estimates (i.e., reproducible results) for 28 practices (44%, 95% CI 31–58%), and inconsistent effect estimates (i.e., non-reproducible results) for 35 practices (56%, 95% CI 42–68%) (Fig. 2). Practices with consistent effects had a smaller number of reproduction attempts per original study than those with inconsistent effects (geometric mean 1.3, 95% CI 1.0–1.6 vs. 1.9, 95% CI 1.4–2.4, *P* = 0.03).

#### Practices with consistent effects

Among 28 practices with consistent effects, most reported lack of efficacy (*n* = 14, 50%, 95% CI 30–69%), with a minority reporting efficacy (*n* = 11, 39%, 95% CI 21–59%) or harm (*n* = 3, 11%, 95% CI 2–28%). Practices consistently reported to be efficacious included lung protective ventilation for acute respiratory distress syndrome (ARDS) and non-invasive ventilation for cardiogenic pulmonary edema (Additional file 1: Table S2). Practices that consistently reported lack of efficacy included immune-modulating therapies for sepsis and continuous (compared with intermittent) renal replacement therapy (Additional file 1: Table S3). The clinical practice with the most consistent evidence of harm was fluid resuscitation with hydroxyethyl starches (Additional file 1: Table S4).

#### Practices with inconsistent effects

For 11 of the 35 practices with inconsistent effects (31%, 95% CI 16–49%), there were multiple different estimates of effect among the reproduction attempts (e.g., original study reports efficacy and some reproduction attempts report lack of efficacy, while others report efficacy) (Additional file 1: Table S5). Of the remaining 24 practices that had one change in the direction of effect between the original study and reproduction attempt, the most common change in effect was from efficacy in the original study to either lack of efficacy or harm in the reproduction attempt (*n* = 14, 58%, 95% CI 36–78%). For four practices, a reproduction attempt reported efficacy after an original study reported lack of efficacy. No reproduction attempt found efficacy for any practice originally found to be harmful.

## Discussion

**Fig. 3:**
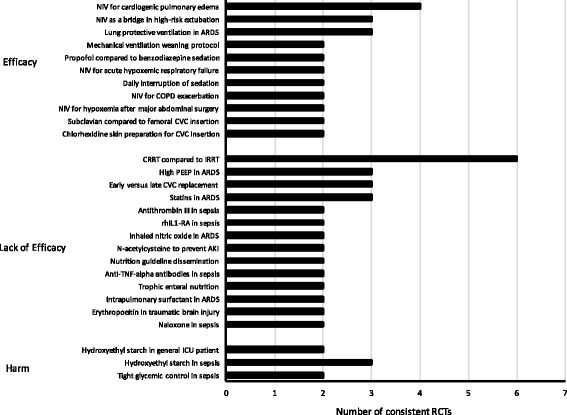
Map of studies with consistent effect estimates between original study and reproduction attempt. Detailed legend: hydroxyethyl starch was examined in both general critically ill and septic patients, thus has duplicate representation within the figure. *AKI* acute kidney injury, *ARDS* acute respiratory distress syndrome, *COPD* chronic obstructive pulmonary disease, *CRRT* continuous renal replacement therapy, *CVC* central venous catheter, *IRRT* intermittent renal replacement therapy, *NIV* non-invasive ventilation, *PEEP* positive end-expiratory pressure, *RCT* randomized clinical trial

We used a rigorous knowledge synthesis method to analyze results reproducibility within a cohort of clinical critical care research published in high-profile journals. The main findings of our study add novel information to this important and evolving scientific area. First, the effects of fewer than half of clinical practices evaluated were assessed for their reproducibility and, of these, less than half had effects that were consistent across original studies and reproduction attempts. Second, slight methodological differences between the original study and corresponding reproduction attempt created challenges reporting reproducibility for certain practices and resulted in most reproduction attempts being an approximate of the corresponding original. Finally, studying results reproducibility within critical care enabled the creation of a map of clinical critical care practices with reproducible evidence (Fig. 3).

Our results compare favorably with prior research [4, 14–18]. Four previous studies examined reproducibility by comparing original studies and reproduction attempts within existing published literature [14–17]. Ioannidis found that 20 (44%) of 45 highly cited studies (at least 1000 indexed citations) claiming a practice to be beneficial, reported results that were consistent with a subsequent reproduction attempt [14]. In two distinct but similar studies, Prasad et al. [15, 16] found that approximately 27% of original research publications in the *New England Journal of Medicine* reported reproduction attempts and, of these, 38–46% found effects that were consistent with the original study. Makel et al. [17] found that 79% of reproduction attempts within published psychology literature reported effects that were consistent with the original study. This estimate decreased to 65% if the authors of the reproduction attempt differed from those of the original study [17]. Two studies examined reproducibility by conducting reproduction attempts for several published original studies [4]. The Open Science Collaboration conducted reproduction attempts for 100 studies published in the psychology literature and found that, depending on the definition of reproducibility, between 36% and 47% of reproduction attempts reported results consistent with the original study [4]. Using a similar approach, Camerer et al. [18] found that, for 18 experimental economic studies, 11 (61%) reproduction attempts found a significant effect in the same direction as the original study.

In conjunction with these previous studies, our study highlights challenges associated with studying reproducibility. First, is the systematic and efficient identification of relevant articles within the vast landscape of published literature. To manage the breadth of the critical care literature, we restricted the primary search to the three general medical journals with the highest impact factors. This was done to reduce the number of early-phase RCTs that are inherently at higher risk for bias, are less relevant to discussions of reproducibility, are more likely published in lower-impact journals, and less likely to influence clinical practice. This restriction may have missed potentially relevant studies. However, articles included in our study are comparable to other reviews of important clinical critical care research [24, 44, 45]. Restricting the primary search to high-profile literature may have overestimated the number of practices with a reproduction attempt. However, through identification of 158 clinical critical care practices, and reporting the estimate of reproduction attempts at the level of the practice rather than the individual original study, it is less likely that inclusion of potentially lower-profile literature within the primary search would considerably alter this estimate. The second challenge associated with examining reproducibility is determining what constitutes a reproduction attempt. There is no consensus definition of a reproduction attempt. Among previous similar studies, definitions are not consistent and are difficult to reliably operationalize [14–17]. In comparison, our definition required greater similarity between original studies and reproduction attempts, with strict criteria pertaining to study design and sample size, and minor latitude given to study population, nature of the intervention and/or control, and primary outcome measure. It is possible that this relatively stricter definition excluded potential reproduction attempts and resulted in a lower estimate of the number of practices with a reproduction attempt. However, by employing a strict definition, our study endeavored to include reproduction attempts that were methodologically similar to the original study and reduced the likelihood that inconsistent results were due to differences in methodological quality [14]. This identifies the third challenge associated with studying reproducibility, which is determining what constitutes a consistent reproduction attempt. Previous studies used conclusions reported by authors to determine whether the results of a reproduction attempt were consistent with the original study [14–17]. We employed a more objective approach that classified the primary efficacy outcome and any pre-specified secondary safety outcome to derive our own assessment of the efficacy of each practice, and used this to determine whether original studies and reproduction attempts reported consistent effects. Accepting the limitations of this approach [11], it is congruent with that employed in previous reproducibility research [14–17], and resulted in a rate of reproducible research that compares favorably with much of the existing clinical literature [4, 14–16].

Our study has implications for clinicians, scientists, and funding agencies. From a clinical perspective, our study may help clinicians interpret the implementation ramifications of experimental critical care research published in high-profile journals. Our results suggest (1) that adoption of practices with one study claiming efficacy should wait until confirmed through a reproduction attempt (e.g., tight glycemic control [46]), (2) that hope not be lost after publication of one study demonstrating lack of efficacy (e.g., prone ventilation [47]), and (3) that clinicians need not wait for a reproduction attempt before deciding against adoption of practices shown to be harmful (e.g., hydroxyethyl starches [48]). Examining reproducibility also enabled the creation of a map of clinical critical care practices with consistent evidence that could broadly inform quality improvement initiatives, such as the Choosing Wisely campaign [49], in deciding what to promote as best practice. The strength of this approach is that it not only includes practices known to have strong reproducible evidence that should be universally adopted (e.g., lung protective ventilation among patients with ARDS) or de-adopted (e.g., hydroxyethyl starch fluid resuscitation), but also less well recognized practices with reproducible evidence that should be adopted (e.g., central venous catheterization via the subclavian compared to jugular or femoral sites) or de-adopted (e.g., high positive end-expiratory pressure in ARDS).

From a scientific perspective, our study demonstrates that understanding which experimental clinical studies require a reproduction attempt, as well as the number of reproduction attempts required for a given clinical practice, requires more study. Due to the risks and costs associated with conducting experimental clinical research, identifying which studies require a reproduction attempt necessitates a thoughtful approach that integrates findings from the original study and factors related to the clinical practice. It also requires a general acceptance within the scientific community of the merit of conducting and publishing the results of reproduction attempts. With regard to findings from the original study, as suggested by our data, wherein no clinical practice found to be harmful in an original study was found to have efficacy in a reproduction attempt, any clinical practice shown to be harmful in a phase III RCT should generally not be examined in additional RCTs. However, among studies reporting efficacy or lack of efficacy, the assessment of whether a reproduction attempt is necessary requires deeper understanding of the likelihood that a reproduction attempt will provide valuable information. If the reproduction attempt is likely to produce consistent results, it is arguably not required, especially if the practice in question is complex and the cost of doing a follow-up RCT is high. On the other hand, if the reproduction attempt is predicted to produce findings that differ from the original study, a reproduction attempt is vitally important. Knowing which studies need a reproduction attempt requires additional understanding of study factors that predict when a reproduction attempt will be consistent with the original study. Such factors include but are not limited to potential small differences in study protocols (i.e., retest versus approximate reproduction attempt), a low fragility index in original studies [50], delta inflation bias in power calculations in reproduction attempts [51], or heterogeneity of treatment effects and the reporting of one effect estimate for a population of patients at differential risk for the outcome [52]. The number of reproduction attempts is also likely an important determinant of consistency, in that as more reproduction attempts are conducted, the likelihood of obtaining a result that differs from the original study increases. The optimal number of reproduction attempts is not clear. When the first reproduction attempt reports findings consistent with the original study, this is likely adequate to assess the efficacy of a given clinical practice, especially if there are no signals from secondary analyses that additional patient subgroups and/or outcomes should be examined. In this case, additional reproduction attempts may result in patients not receiving beneficial practices (or unnecessarily experiencing ineffective practices), and waste of valuable healthcare and scientific resources. When the findings from a first reproduction attempt are not consistent with the original study, clinicians and scientists should view that inconsistency as an opportunity to pause and re-examine each component of the clinical question (i.e., population, intervention, etc.) before moving forward with any additional experimental research. Additional understanding pertaining to rates and predictors of reproducibility will help scientists decide which practices warrant repeat examination through a reproduction attempt, and may help design studies that are less susceptible to non-reproducibility. Similarly, funding agencies may be better positioned to weigh the relative importance and methodological strength of a proposed reproduction attempt, which may help inform the controversial balance between funding science that intends to examine existing concepts and science that intends to discover new concepts.

## Conclusions

Fewer than half of clinical critical care practices with research published in high-profile journals were evaluated for reproducibility and, of these, less than half had reproducible effects. Heterogeneity within study populations and delivery of interventions presents challenges to studying reproducibility within clinical research. These challenges notwithstanding, implications of our work include that caution is warranted when interpreting initial reports of clinical research; specialty societies should consider waiting for evidence of reproducibility before defining best practices given the potential broad impact of their recommendations. Further, researchers and funding agencies should increase efforts to evaluate the reproducibility of clinical experiments, with examination of scientific reproducibility being an accepted and required part of scientific discourse.

## Additional file

Additional file 1: **Table** **S1.** Clinical practices without a reproduction attempt. **Table S2.** Clinical practices with consistent estimates of efficacy between original studies and reproduction attempts. **Table S3.** Clinical practices with consistent estimates of lack of efficacy between original studies and reproduction attempts. **Table S4.** Clinical practices with consistent estimates of harm between original studies and reproduction attempts. **Table S5.** Clinical practices with inconsistent effect estimates between original studies and reproduction attempts. **Figure S1.** Flow diagram showing study design including electronic search strategy, article eligibility criteria, and reproducibility classification. **Figure S2.** The relationship between time since publication of the original study and the occurrence of a first reproduction attempt. **Online Appendix.** MEDLINE Search Strategy (April 4, 2016). (DOCX 1175 kb)

## Abbreviations

ACP: American College of Physicians
AKI: acute kidney injury
ARDS: acute respiratory distress syndrome
CI: confidence interval
COPD: chronic obstructive pulmonary disease
CRRT: continuous renal replacement therapy
CVC: central venous catheter
ICU: intensive care unit
IRRT: intermittent renal replacement therapy
NIV: non-invasive ventilation
PEEP: positive end-expiratory pressure
PRESS: peer review of electronic search strategies
RCT: randomized controlled trial
VAP: ventilator-associated pneumonia
