# Supplementary material for: Reproducibility of clinical research in critical care: a scoping review
Source: BMC Med. 2018 Feb 21;16:26. doi: 10.1186/s12916-018-1018-6 (PMC5820784; doi:10.1186/s12916-018-1018-6)
Supplement: Supplementary file 1 — Table S1. Clinical practices without a reproduction attempt. Table S2. Clinical practices with consistent estimates of efficacy between original studies and reproduction attempts. Table S3. Clinical practices with consistent estimates of lack of efficacy between original studies and reproduction attempts. Table S4. Clinical practices with consistent estimates of harm between original studies and reproduction attempts. Table S5. Clinical practices with inconsistent effect estimates between original studies and reproduction attempts. Figure S1. Flow diagram showing study design including electronic search strategy, article eligibility criteria, and reproducibility classification. Figure S2. The relationship between time since publication of the original study and the occurrence of a first reproduction attempt. Online Appendix. MEDLINE Search Strategy (April 4, 2016). (DOCX 1175 kb) [file 12916_2018_1018_MOESM1_ESM.docx]

**Additional file 1**

Supplement to: Niven DJ, McCormick TJ, Straus SE, Hemmelgarn BR, Jeffs L, Barnes TRM, Stelfox HT. Reproducibility of Clinical Research in Critical Care: A Scoping Review

**Table S1.** Clinical practices without a reproduction attempt.

**Table S2.** Clinical practices with consistent estimates of efficacy between original studies and reproduction attempts.

**Table S3.** Clinical practices with consistent estimates of lack of efficacy between original studies and reproduction attempts.

**Table S4.** Clinical practices with consistent estimates of harm between original studies and reproduction attempts.

**Table S5.** Clinical practices with inconsistent effect estimates between original studies and reproduction attempts.

**Figure S1.** Flow diagram showing study design including electronic search strategy, article eligibility criteria, and reproducibility classification.

**Figure S2.** The relationship between time since publication of the original study and the occurrence of a first reproduction attempt.

**Online Appendix** – MEDLINE Search Strategy (April 4, 2016)

**Online-only References**

**Table S1. Clinical practices without a reproduction attempt.**

| **Condition** | **Clinical Practice** | **Study** | **Primary**  **Outcome** | **Effect of Clinical Practice** |
| --- | --- | --- | --- | --- |
| **Practices with Evidence of Efficacy** | |  |  |  |
| ***General critical illness*** | Restrictive transfusion of red blood cells (hemoglobin target ≥ 70 g/L) | Hebert, 1999[1] | Mortality – 30 day | No difference in 30-day mortality, but decrease risk of death in hospital (ARR 5.9%, *P* = .05) |
|  | Supplemental parenteral nutrition | Heidegger, 2013[2] | Nosocomial infections | Decrease in risk of nosocomial infection (HR 0.65, 95% CI 0.43-0.97, *P* = .03) |
| ***Mechanical ventilation (excluding ARDS)*** | Use of positive end-expiratory pressure when weaning patients from mechanical ventilation | Feeley, 1975[3] | None reported | Improvement in oxygenation during weaning |
|  | Physiotherapy schedule to coincide with daily interruption of sedation | Schweickert, 2009[4] | Independent functional status at hospital discharge | Increase in likelihood of returning to independent functional status at hospital discharge (OR 2.70, 95% CI 1.2-6.1, *P* = .02) |
|  | Dexmedetomidine versus lorazepam | Pandharipande, 2007[5] | Days alive without delirium or coma | Increase in number of days alive without delirium or coma (7 vs 3 days, *P* = .01) |
|  | No sedation | Strom, 2010[6] | Ventilator-free days | Increase in number of ventilator-free days (4.20 days, *P* = .02) |
|  | Patient-directed music | Chlan, 2013[7] | Anxiety on visual-analog scale | Decrease in anxiety and required amount of sedation |
|  | Weaning via early tracheostomy collar (compared pressure support ventilation) | Jubran, 2013[8] | Time to wean from mechanical ventilation | Decrease in time to weaning from mechanical ventilation (HR 1.43, 95% CI 1.03-1.98, *P* = .03) |
| ***Respiratory failure (not ventilated)*** | Ofloxacin during an acute exacerbation of COPD | Nouira, 2001[9] | Mortality - hospital | Decrease in risk of death in hospital (ARR 17.5%, 95% CI 4.3-30.7%) |
|  | Systemic corticosteroids in severe community-acquired pneumonia | Torres, 2015[10] | Treatment failure | Decrease risk of treatment failure (ARR 18%, OR 0.34, 95% CI 0.14 to 0.87, *P* = 0.02) |
|  | High-flow nasal oxygen (compared to non-invasive ventilation) after cardiac surgery | Stephan, 2015[11] | Treatment failure | High-flow nasal oxygen non-inferior to non-invasive ventilation (absolute difference 0.9%, 95% CI -4.9 to 6.6%, *p* = 0.003) |
|  | High-flow nasal oxygen (compared to standard oxygen) in low-risk patients post-extubation | Hernandez, 2016[12] | Reintubation (72 hours) | Decrease risk of reintubation at 72 hours post-extubation (ARR 7.2%, 95% CI 2.5% to 12.2%, *P* = 0.004) |
| ***Sepsis*** | Naloxone infusion | Roberts, 1988[13] | Not reported | Decrease in ionotrope and vasopressor doses in naloxone group at 8 and 16 hours |
| ***Neurological*** |  |  |  |  |
| Brain death | Desmopressin for diabetes insipidus | Guesde, 1998[14] | Renal replacement therapy in recipient | Decrease in amount of diuresis with no impact on renal replacement therapy in recipient (23% vs 20%) |
|  | Hypothermia (compared to normothermia) in organ donors | Niemann, 2015[15] | Delayed renal graft function | Decrease risk in delayed renal graft function (ARR 11%, OR 0.62, 95% CI 0.42 to 0.92, *P* = 0.02) |
| Traumatic brain injury | Phenytoin to prevent the development of seizures | Temkin, 1990[16] | Seizures | Decrease in risk for seizures (RR 0.27, 95% CI 0.12-0.62) |
| ***Nosocomial complications*** |  |  |  |  |
| Ventilator-associated pneumonia | Antibiotic duration: 8 versus 15 days | Chastre, 2003[17] | Mortality – 28 day | Decrease in antibiotic use with no untoward effect on survival (risk difference 1.6%, 90% CI -3.7% to 6.9%) |
|  | Silver-coated endotracheal tubes | Kollef, 2008[18] | Ventilator-associated pneumonia | Decrease in ventilator-associated pneumonia (RRR 35.9%, 95% CI 3.6-69.0%, *P* = .03) |
| **Condition** | **Clinical Practice** | **Study** | **Primary**  **Outcome** | **Effect of Clinical Practice** |
| Catheter-related bloodstream infection | Central venous catheter with subcutaneous cuff | Flowers, 1989[19] | Catheter colonization rate | Decrease in risk of catheter colonization (ARR 26.8%, *P* = .02) |
|  | Antibiotic bonded central venous catheter | Kamal, 1991[20] | Catheter-related complications | Decrease in risk of catheter-related complications (ARR 12%, *P* = .004) |
|  | Skin preparation with chlorhexidine (2%) prior to central venous catheter insertion (compared to 70% alcohol, or 10% povidone-iodine) | Maki, 1991[21] | Catheter-related bloodstream infection | Decrease in catheter-related bloodstream infection  Chlorhexidine: 2.3 per 100 catheters  Povidone: 9.3 per 100 catheters  Alcohol: 7.1 per 100 catheters |
|  | Chlorhexidine impregnated sponge for central venous catheter dressing change | Timsit, 2009[22] | Major catheter-related infection | Decrease in risk of major catheter-related infection (HR 0.39, 95% CI 0.17-0.93, *P* = .03) |
| Nosocomial infection (general) | Universal decolonization (compared to selective decolonization compared to standard practice of screening and isolation) | Huang, 2013[23] | MRSA clinical culture | Decrease in MRSA clinical isolates  Universal decolonization: HR 0.63 (95% CI 0.52-0.75)  Targeted decolonization: HR 0.75 (0.63-0.89) |
| Postoperative infection | Anti-endotoxin antibody | Baumgartner, 1985[24] | None reported | Decrease in risk of death in hospital (1.6% vs 6.6%) |
|  | Intravenous immunoglobulin | Intravenous Immunoglobulin Collaborative Study Group, 1992[25] | Mortality - hospital | Decrease in incidence of post-operative infections |
| Gastrointestinal bleeding | Antacid compared to no treatment | Hastings, 1978[26] | Gastrointestinal bleeding | Decrease risk of gastrointestinal bleed (ARR 20.5%, *P* = 0.005) |
| Pressure ulcer | Air suspension bed | Inman, 1993[27] | Pressure ulcer | Decrease in risk of developing a pressure ulcer (16.3% vs 79.5%) |
| ***Acute kidney injury*** | Daily intermittent hemodialysis (compared to conventional hemodialysis schedule) | Schiffl, 2002[28] | Survival – 14 days after last treatment | Increase in survival 14 days after last run of hemodialysis (28% vs 46%, *P* = 0.01) |
| ***General resuscitation*** |  |  |  |  |
| Cardiac arrest | Cardiopulmonary resuscitation with interposed abdominal counterpulsation | Sack, 1992[29] | Return of spontaneous circulation | Increased likelihood of achieving return of spontaneous circulation (ARR 24%, *P* = .007) |
|  | Active compression-decompression cardiopulmonary resuscitation | Cohen, 1993[30] | Survival – 24 hours | Increased likelihood of survival to 24 hours (ARR 36%, *P* = .004) |
| ***Trauma*** | Glutamine supplementation | Houdijk, 1998[31] | Infection – 15 days after randomization | Decrease in risk of infection 15 days after study entry (ARR 26%, *P* = .02) |
|  | Systemic mineralocorticoid | Roquilly, 2011[32] | Hospital-acquired pneumonia | Decrease in risk of hospital-acquired pneumonia (HR 0.51, 95% CI 0.30-0.83, *P* = .007) |
| ***Other*** |  |  |  |  |
| Phenobarbitol toxicity | Multi-dose activated charcoal | Pond, 1984[33] | None reported | Decrease in half-life of phenobarbital |
| Severe meliodosis | Early ceftazidime | White, 1989[34] | Mortality | Decrease in risk of death (ARR 37%, *P* = .009) |
|  |  |  |  |  |
| Necrotizing pancreatitis | Early cefuroxime | Sainio, 1995[35] | Mortality - hospital | Decrease in risk of death in hospital (ARR 20%, *P* = .03) |
| **Practices with Evidence of Lack of Efficacy** | |  |  |  |
| ***General critical illness*** | Dichloroacetate for acidosis | Stacpoole, 1992[36] | Mortality – 24 hour | No difference in survival to 24 hours (54% vs 59%) |
|  | Albumin versus crystalloid | Finfer, 2004[37] | Mortality – 28 day | No difference in risk of death at 28 days (RR 0.99, 95% CI 0.91-1.09) |
|  | Colloid versus crystalloid | Annane, 2013[38] | Mortality – 28 day | No difference in risk of death at 28 days (RR 0.96, 95% CI 0.88-1.04) |
| **Condition** | **Clinical Practice** | **Study** | **Primary**  **Outcome** | **Effect of Clinical Practice** |
|  | Early parenteral nutrition in patients with contraindication to enteral nutrition^a^ | Doig, 2013[39] | Mortality – 60 day | No difference in risk of death at 60 days (21.5% vs 22.8%, *P* = .60) |
|  | Early parenteral nutrition (compared to enteral nutrition) | Harvey, 2014[40] | Mortality – 30 day | No difference in risk of death at 30 days (RR 0.97, 95% CI 0.86 to 1.08, *P* = .57) |
|  | High-dose Vitamin D_3_ (compared to placebo) | Amrein, 2014[41] | Hospital length of stay | No difference in hospital length of stay (Vitamin D_3_ 20.1 days (IQR 11.1 – 33.3 days) vs placebo 19.3 days (IQR 11.1 – 34.9 days)) |
|  | Buffered crystalloid versus saline for fluid resuscitation | Young, 2015[42] | Acute kidney injury | No difference in acute kidney injury (RR 1.04, 95% CI 0.80 to 1.36, *P* = 0.77) |
|  |  |  |  |  |
|  | Fresh versus standard aged red blood cell transfusion | Lacroix, 2015[43] | Mortality – 90 day | No difference in risk of death at 90 days (37.0% vs 35.3%, ARR -1.7%, 95% CI -5.5 to 2.1%) |
| ***ARDS*** | High dose systemic glucocorticoids | Bernard, 1987[44] | Mortality – 45 days | No difference in risk of death at 45 days (ARR 3%, *P* = .74) |
|  | Aerosolized protein-free surfactant | Anzueto, 1996[45] | Mortality – 30 days | No difference in risk of death at 30 days (ARR 0%) |
|  | Tailored tidal volume and positive end-expiratory pressure | Ranieri, 1999[46] | Change in cytokine concentrations | No difference in any measured cytokines |
|  | Ketoconazole | ARDS Clinical Trials Network, 2000[47] | Mortality – hospital | No difference in risk of death in hospital (ARR -1.1%, *P* = .85) |
|  | Conservative versus liberal fluid strategy | ARDS Clinical Trials Network, 2006[48] | Mortality – 60 days | No difference in risk of death at 60 days (ARR 2.9%, 95% CI -2.6 to 8.4%) |
|  | Trophic enteral nutrition | ARDS Clinical Trials Network, 2012[49] | Ventilator-free days | No difference in ventilator-free days (difference -0.2 days, 95% CI -1.4 to 1.2 days, *P* = 0.89) |
| ***Mechanical ventilation (excluding ARDS)*** | Early use of positive end-expiratory pressure in those at risk for ARDS | Pepe, 1984[50] | Acute respiratory distress syndrome | No difference in risk of developing ARDS (25% vs 27%) |
|  | Daily sedative interruption vs protocolized sedation | Mehta, 2012[51] | Time to successful extubation | No difference in time to successful extubation (HR 1.08, 95% CI, 0.86-1.35, *P* = .52) |
|  | Prone position in acute hypoxemic respiratory failure | Guerin, 2004[52] | Mortality – 28 day | No difference in risk of death at 28 days (RR 0.97, 95% CI 0.79-1.19, *P* = .77) |
|  | Immunonutrition | Heyland, 2013[53] | Mortality – 28 day | No difference in risk of death at 28 days (OR 1.09, 95% CI 0.86-1.40) |
|  |  | Van Zanten, 2014[54] | Nosocomial infection | No difference in risk of nosocomial infection (53% vs 52%, *P* = .96) |
|  | Acetazolamide in patients with COPD | Faisy, 2016[55] | Duration of mechanical ventilation | No difference in duration mechanical ventilation (absolute difference -16.0 hours, 95% CI -36.5 to 4.0, *P* = 0.17) |
| ***Respiratory failure (not ventilated)*** | Respiratory stimulants | Edwards, 1967[56] | None reported | No difference in reversal of respiratory failure |
|  | CPAP for hypoxemic respiratory failure | Delclaux, 2000[57] | Intubation | No difference in risk of intubation (34% vs 39%, *P* = .53) |
|  | Etomidate versus ketamine for induction of intubation | Jabre, 2009[58] | SOFA difference – first 3 days in ICU | No difference in SOFA score during first 3 days of ICU admission (0.70, 95% CI 0.0-1.4, *P* = .06) |
|  | High-flow nasal cannula (HFNC) (versus standard oxygen or non-invasive ventilation) in hypoxemic respiratory failure | Frat, 2015[59] | Intubation – day 28 | No difference in intubation rate (HFNC 38%, standard oxygen 47%, non-invasive ventilation 50%, *P* = 0.18) |
| ***Sepsis*** | Ibuprofen | Bernard, 1997[60] | Mortality – 30 day | No difference in risk of death at 30 days (37% vs 40%, *P* = NS) |
| **Condition** | **Clinical Practice** | **Study** | **Primary**  **Outcome** | **Effect of Clinical Practice** |
|  | Bradykinin antagonist | Fein, 1997[61] | Mortality – 28 day | No difference in risk of death at 28 days (40.1% vs 40.3%) |
|  | Tifacogin | Abraham, 2003[62] | Mortality – 28 day | No difference in risk of death at 28 days (34.2 vs 33.9%, *P* = .75) |
|  | Fludricortisone and hydrocortisone versus hydrocortisone alone | Annane, 2010[63] | Mortality – hospital | No difference in risk of death in hospital (RR 0.94, 95% CI 0.77-1.14, *P* = .5) |
|  | Eritoran | Opal, 2013[64] | Mortality – 28 day | No difference in risk of death at 28 days (HT 1.05, 95% CI 0.88-1.26, *P* = .59) |
|  | Moxifloxacin and meropenem versus meropenem alone | Brunkhorst, 2012[65] | Mean SOFA over first 14 days | No difference in severity of illness scores during first 14 days (8.3 vs 7.9, *P* = .36) |
|  | Epinephrine versus norepinephrine/dobutamine | Annane, 2007[66] | Mortality – 28 day | No difference in risk of death at 28 days (40% vs 34%, *P* = .31) |
|  | Vasopressin versus norepinephrine | Russell, 2008[67] | Mortality – 28 day | No difference in risk of death at 28 days (35.4% vs 39.3%, *P* = .26) |
|  | Albumin (20%) versus crystalloid resuscitation | Caironi, 2014[68] | Mortality – 28 day | No difference in risk of death at 28 days (RR 1.0, 95% CI 0.87-1.14, *P* = .94) |
|  | Mean arterial pressure target 80 – 85 mmHg versus 60 – 65 mmHg | Asfar, 2014[69] | Mortality – 28 day | No difference in risk of death at 28 days (HR 1.07, 95% CI 0.84-1.38, *P* = .57) |
|  | Acetaminophen (versus placebo) for fever | Young, 2015[70] | ICU-free days to day 28 | No difference in ICU-free days (absolute difference 0 days, 96.2% CI 0 – 1 days, *P* = 0.07) |
|  | Restrictive compared to liberal hemoglobin transfusion in septic shock | Holst, 2014[71] | Mortality – 90 days | No difference in risk of death at 90 days (RR 0.94, 95% CI 0.78 to 1.09, *P* = 0.44) |
| ***Neurological*** |  |  |  |  |
| Traumatic brain injury | Pegorgotein | Young, 1996[72] | Glasgow outcome scale – 3 months | No difference in likelihood of good neurological recovery at 3 months (low dose 28% vs high dose 25% vs placebo 27%) |
| Anoxic encephalopathy | Thiopental loading | Brain Resuscitation Clinical Trial I Study Group, 1986[73] | Good cerebral recovery | No difference in likelihood of good cerebral recovery (20% vs 15%) |
|  | Nimodipine | Roine, 1990[74] | Mortality – 1 year | No difference in risk of death at one year (40% vs 36%) |
|  | Nimodipine | Roine, 1993[75] | Cognitive deficits – 6 months | No difference in likelihood of cognitive deficits (31% vs 48%) |
|  | Lidoflazine | Brain Resuscitation Clinical Trial II Study Group, 1991[76] | Mortality – 6 months | No difference in risk of death at 6 months (82% vs 83%) |
| Status epilepticus | Lorazepam versus diazepam | Leppik, 1983[77] | None reported | No difference in likelihood of seizure control (89% vs 76%) |
| ***Nosocomial complications*** |  |  |  |  |
| Ventilator-associated pneumonia | Simvastatin | Papazian, 2013[78] | Mortality – 28 day | No difference in risk of death at 28 days (HR 1.45, 95% CI 0.83-2.51, *P* = .10) |
| Catheter-related bloodstream infection | Central venous catheter dressing change every 7 days versus 3 days | Timsit, 2009[22] | Catheter colonization | Non-inferiority of 7-day versus 3-day changes (HR 0.99, 95% CI 0.77-1.28) |
| Nosocomial colonization with antibiotic-resistant bacteria | Aggressive surveillance and isolation | Huskins, 2011[79] | Incidence MRSA or VRE clinical isolate | No difference in incidence of MRSA/VRE clinical isolates (40.4 vs 35.6/1000 patient-days, *P* = .35) |
|  | Universal gown and glove for all patients | Harris, 2013[80] | Acquisition of MRSA or VRE | No difference in risk of nosocomial colonization with MRSA or VRE (absolute difference -1.71 acquisitions per 1000 person-days, *P* = .57) |
| **Condition** | **Clinical Practice** | **Study** | **Primary**  **Outcome** | **Effect of Clinical Practice** |
| Venous thromboembolism prophylaxis | Dalteparin versus unfractionated heparin | PROTECT Investigators, 2011[81] | Proximal leg deep vein thrombosis | No difference in risk of proximal leg deep vein thrombosis (HR 0.92, 95% CI 0.68-1.23, *P* = .57) |
| ***Acute kidney injury*** | Anaritide | Allgren, 1997[82] | Survival-free of renal replacement therapy – 21 days | No difference in survival-free of renal replacement therapy at 21 days (43% vs 47%, *P* = .35) |
|  | Renal-dose dopamine | Bellomo, 2000[83] | Peak creatinine during study drug | No difference in peak creatinine during study drug administration (245 vs 249 μmol/L, *P* = .93) |
|  | Peritoneal dialysis | Phu, 2002[84] | Normalization of creatinine | No difference in rate of normalization of creatinine |
|  | High intensity renal replacement therapy | VA/NIH Acute Renal Failure Trial Network, 2008[85] | Mortality – 60 day | No difference in risk of death at 60 days (OR 1.09, 95% CI 0.86-1.40, *P* = .47) |
| ***General resuscitation*** |  |  |  |  |
| Cardiac arrest | Mechanical versus manual cardiopulmonary resuscitation | Taylor, 1978[86] | Survival to 24 hours | No difference in likelihood of survival to 24 hours (42% vs 38%) |
|  | Intravenous magnesium | Thel, 1997[87] | Return of spontaneous circulation | No difference in likelihood of achieving return of spontaneous circulation (54% vs 60%, *P* = .44) |
|  | High dose epinephrine | Stiell, 1992[88] | Return of spontaneous circulation for 1 hour | No difference in likelihood of experiencing return of spontaneous circulation for 1 hour (18% vs 23%, *P* = .12) |
|  | Vasopressin versus epinephrine | Stiell, 2001[89] | Survival 1 hour after stopping resuscitation | No difference in survival 1 hour after acute resuscitation stopped (39% vs 35%, *P* = .66) |
| **Practices with Evidence of Harm** | |  |  |  |
| ***General critical illness*** | Early parenteral nutrition^a^ | Casaer, 2011[90] | Length of stay - ICU | Increase in risk of infection and slower overall recovery (HR 1.06, 95% CI 1.00-1.13, *P* = .04) |
|  | Human growth hormone supplementation | Takala, 1999[91] | Length of stay - ICU | Increase in ICU length of stay in both RCTs reported in this article |
|  |  |  |  |  |
| ***ARDS*** | Intravenous salbutamol | Gao Smith, 2012[92] | Mortality – 28 day | Increase in risk of death at 28 days (RR 1.47, 95% CI 1.03-2.08) |
| ***Mechanical ventilation (excluding ARDS)*** | Intravenous glutamine | Heyland, 2013[53] | Mortality – 28 day | Increase in risk of death at 28 days (OR 1.28, 95% CI 1.00-1.64, *P* = .05) |
| ***Sepsis*** | Epinephrine versus dopamine | Day, 1996[93] | Lactate concentration | Worsening lactic acidosis with epinephrine (+3.2 mmol/L vs -1.0 mmol/L) |
| ***Neurological*** |  |  |  |  |
| Delirium | Rivastigmine | Van Eijk, 2010[94] | Delirium duration | Study stopped early due to increase in risk of death (22% vs 8%, *P* = .07) |
|  | Transfusion threshold 100g/L compared to 70g/L | Robertson, 2014[95] | Glasgow outcome scale – 6 months | No difference in neurological outcome, but Increase risk of thromboembolic events with higher transfusion threshold (21.8% vs 8.1%, *P* = .009) |
| Bacterial meningitis | Hypothermia | Mourvillier, 2013[96] | Glasgow outcome scale – 3 months | Study stopped early due to increase in risk of death (RR 1.99, 95% CI 1.05-3.77, *P* = .04) |
| ***Acute kidney injury*** | Atorvastatin versus placebo following cardiac surgery | Billings, 2016[97] | Acute kidney injury | No overall difference in acute kidney injury (RR 1.06, 95% CI 0.78 – 1.46, *P* = 0.75);study stopped early due to increased acute kidney injury in patients with chronic kidney disease |
| **Condition** | **Clinical Practice** | **Study** | **Primary**  **Outcome** | **Effect of Clinical Practice** |
| ***Trauma*** | Diaspirin cross-linked hemoglobin | Sloan, 1999[98] | Mortality – 28 day | Increase in risk of death at 28 days (46% vs 17%, *P* = .003) |

ARR = absolute risk reduction; ARDS = acute respiratory distress syndrome; COPD = chronic obstructive pulmonary disease; HR = hazard ratio; ICU = intensive care unit; MRSA = methicillin-resistant *Staphylococcus aureus*; OR = odds ratio; RR = risk ratio; RRR = relative risk reduction; SOFA = sepsis-related organ failure assessment; VRE= vancomycin-resistant enterococcus

**Table S2. Clinical practices with consistent estimates of efficacy between original studies and reproduction attempts.**

| **Condition** | **Clinical Practice** | **Study** | **Type of Study** | **Primary**  **Outcome** | **Effect of Clinical Practice** |
| --- | --- | --- | --- | --- | --- |
| ***ARDS*** | Lung protective mechanical ventilation | Amato, 1998[99] | Original | Mortality – 28 day | Decrease in risk of death at 28 days (ARR 33%, *P* < .001) |
|  |  | Brower, 2000[100] | Reproduction attempt | Mortality - hospital | Decrease in risk of death in hospital (ARR 8.8%, *P* = .007) |
|  |  | Villar, 2006[101] | Reproduction attempt | Mortality - ICU | Decrease in risk of death in ICU (ARR 21.3%, *P* = .04) |
|  |  |  |  |  |  |
| ***Mechanical ventilation (excluding ARDS)*** | Propofol versus midazolam | Aitkenhead, 1989[102] | Original | Not reported | Improvement in time to wean from ventilation and recover consciousness |
|  |  | Chamorro, 1996[103] | Reproduction attempt | Not reported | Improved ventilator synchrony and time to wakefulness with propofol |
|  | Daily sedative interruption | Kress, 2000[104] | Original | Duration of mechanical ventilation | Decrease in duration of mechanical ventilation (4.9 vs 7.3 days, *P* = .004) |
|  |  | Girard, 2008[105] | Reproduction attempt | Time breathing without assistance | Paired spontaneous awakening and spontaneous breathing trial increased time without assisted breathing (3.1 days, 95% CI 0.7 – 5.6, *P* = .02) |
|  | Mode of weaning – Pressure support ventilation (PSV), T-piece, or Synchronized Intermittent Mandatory Ventilation (SIMV) | Brochard, 1994[106] | Original | Weaned from mechanical ventilation – day 21 | Increase in likelihood of weaning with PSV compared to T-piece, or SIMV (PSV 77% vs T-piece 57% vs SIMV 58%, *P* = .05) |
|  | Modes of weaning – IMV, PSV, SBT ≥ 2 times per day, SBT once per day | Esteban, 1995[107] | Reproduction attempt | Weaning from mechanical ventilation | Greatest likelihood of weaning associated with once daily SBT  Compared to IMV: HR 2.83 (1.36-5.89)  Compared to PSV: HR 2.05 (1.04-4.04)  Compared to multiple daily SBT: HR 1.24 (0.64 – 2.41) |
| ***Respiratory failure (not ventilated)*** | Non-invasive ventilation (NIV) in respiratory failure (general) | Kramer, 1995[108] | Original | Intubation | Decrease in risk of requiring intubation (ARR 42%, *P* = .049) |
|  |  | Antonelli, 1998[109] | Reproduction attempt | Improvement in P/F ratio | Improvement in oxygenation (62% vs 47%, *P* = .21) |
|  | NIV as a bridge in high risk extubations | Nava, 2005[110] | Original | Intubation | Decrease in risk of requiring re-intubation (ARR 16.2%, *P* = .027) |
|  |  | Ferrer, 2006[111] | Reproduction attempt | Respiratory failure after extubation | Decrease in risk of developing recurrent respiratory failure (ARR 17%, *P* = .029) |
|  |  | Ferrer, 2009[112] | Reproduction attempt | Respiratory failure 72 hours after extubation | Decrease in risk of developing recurrent respiratory failure (OR 5.32, 95% CI 2.11 – 13.46, *P* < .0001) |
| **Condition** | **Clinical Practice** | **Study** | **Type of Study** | **Primary**  **Outcome** | **Effect of Clinical Practice** |
|  | NIV in cardiogenic pulmonary edema | Bersten, 1991[113] | Original | Intubation | Decrease in risk of requiring intubation (ARR 35%, *P* = .005) |
|  |  | Masip, 2000[114] | Reproduction attempt | Intubation | Decrease in risk of requiring intubation (ARR 28%, *P* = .037) |
|  |  | Nava, 2003[115] | Reproduction attempt | Intubation | Decrease in risk of requiring intubation among patients with hypercarbia (ARR 23%, *P* = .015) |
|  |  | Park, 2004[116] | Reproduction attempt | Intubation | Decrease in risk of requiring intubation (ARR 35%, *P* = .001) |
|  | NIV in acute exacerbation of COPD | Bott, 1993[117] | Original | Not reported | Improvement in arterial blood gas pH after one hour of therapy |
|  |  | Brochard, 1995[118] | Reproduction attempt | Intubation | Decrease in risk of requiring intubation (ARR 48%, *P* < .001) |
|  | NIV post-abdominal surgery | Squadrone, 2005[119] | Original | Intubation | Decrease in risk of requiring intubation (RR 0.099, 95% CI 0.01-0.76, *P* = .005) |
|  |  | Jaber, 2016[120] | Reproduction attempt | Intubation – 7 days | NIV, 33.1% versus standard oxygen, 45.5%; ARR 12.4% (95% CI 1.3 to 23.5%, *p* = 0.03) |
| ***Nosocomial complications*** | |  |  |  |  |
| Central venous catheter complications | Anti-septic: chlorhexidine gluconate versus povidone iodine | Mimoz, 1996[121] | Original | Catheter colonization | Decrease in risk of catheter colonization (RR 0.4, 95% CI 0.1 to 0.9, *P* < .01) |
|  |  | Mimoz, 2015[122] | Reproduction attempt | Catheter-related infections | Decrease in risk of catheter-related infections (HR 0.15, 95% CI 0.05 – 0.41, *P* = .0002) |
|  | Insertion site: subclavian versus femoral | Merrer, 2001[123] | Original | Catheter-related complications | Decrease in risk of infectious and thrombotic complications with subclavian site (ARR 33%, 95% CI 23-43%) |
|  | Insertion site: internal jugular versus femoral | Parienti, 2008[124] | Original | Catheter colonization at removal | No difference in risk of catheter colonization (HR 0.85, 95% CI 0.62-1.16, *P* = .31) |
|  | Insertion site: subclavian versus internal jugular versus femoral | Parienti, 2015[125] | Reproduction attempt | Composite – CRBSI and DVT | Femoral vs subclavian: HR 3.5 (95% CI 1.5-7.8)  Jugular vs subclavian: HR 2.1 (95% CI 1.0-4.3)  Femoral vs jugular: HR 1.3 (95% CI 0.8-2.1) |

ARR = absolute risk reduction; ARDS = acute respiratory distress syndrome; COPD = chronic obstructive pulmonary disease; CRBSI = catheter-related bloodstream infection; CPAP = continuous positive airway pressure; DVT = deep vein thrombosis; HR = hazard ratio; ICU = intensive care unit; NIV = non-invasive ventilation; OR = odds ratio; RR = risk ratio.

Excluded from this table was the ACURYSYS RCT that showed that early use of cisatracurium reduced the risk of death at 90 days in patients with ARDS (HR 0.68, 95% CI 0.48-0.98, *P* = .04)[126], as it’s not clear that this will be a consistent result until the results of its reproduction attempt [127] are published.

**Table S3. Clinical practices with consistent estimates of lack of efficacy between original studies and reproduction attempts.**

| **Condition** | **Clinical Practice** | **Study** | **Type of Study** | **Primary Outcome** | **Effect of Clinical Practice** |
| --- | --- | --- | --- | --- | --- |
| ***General critical illness*** | Active nutrition guideline dissemination | Jain, 2006[128] | Original | Nutritional adequacy | No difference in likelihood of achieving nutritional adequacy (8% vs 6.2%, *P* = .54) |
|  |  | Doig, 2008[129] | Reproduction attempt | Mortality - hospital | No difference in risk of death in hospital (28.9% vs 27.4%, *P* = .75) |
| ***ARDS*** | High positive end-expiratory pressure | Brower, 2004[130] | Original | Mortality – hospital | No difference in risk of death in hospital (ARR 2.6%, 95% CI -10.0 to 4.7%) |
|  |  | Mercat, 2008[131] | Reproduction attempt | Mortality – 28 day | No difference in risk of death at 28 days (RR 1.12, 95% CI 0.90-1.40) |
|  |  | Meade, 2008[132] | Reproduction attempt | Mortality – hospital | No difference in death in hospital (RR 0.90, 95% CI 0.77-1.05) |
|  | Inhaled nitric oxide | Lundin, 1999[133] | Original | Reversal of acute lung injury | No difference in frequency of reversal of acute lung injury (61% vs 54%, *P* = .2) |
|  |  | Taylor, 2004[134] | Reproduction attempt | Ventilator-free days | No difference in ventilator-free days (-0.1 days, 95% CI -2.0 to 1.9) |
|  | Recombinant surfactant protein C-based surfactant | Spragg, 2004[135] | Original | Ventilator-free days | No difference ventilator-free days (RR 0.94, 95% CI 0.78-1.13) |
|  |  | Spragg, 2011[136] | Reproduction attempt | Mortality – 28 day | No difference in risk of death at 28 days (22.7% vs 23.8%, *P* = .26) |
|  | Full versus trophic enteral nutrition | Rice, 2011[137] | Original | Ventilator-free days | No difference in ventilator-free days (-3.2 days, 95% CI -5.8 to -0.7 days, *P* = .02) |
|  |  | Arabi, 2015[138] | Reproduction attempt | Mortality – 90 day | No difference in risk of death at 90 days (RR 0.94, 95% CI 0.76 – 1.16) |
|  | Statins | Craig, 2011[139] | Original | Extravascular lung water | No difference in extravascular lung water (13.7 vs 13.4, *P* = .90) |
|  |  | ARDS Clinical Trials Network, 2014[140] | Reproduction attempt | Mortality – 60 day | No difference in 60-day mortality (28.5% vs 24.9%, *P* = .21) |
|  |  | McAuley, 2014[141] | Reproduction attempt | Ventilator-free days to day 28 | No difference in ventilator-free days (12.6 days vs 11.5 days, *p* = 0.21) |
| ***Sepsis*** | Antithrombin III | Baudo, 1998[142] | Original | Mortality | No difference in risk of death (50% vs 46%, P = NS) |
|  |  | Warrern, 2001[143] | Reproduction attempt | Mortality – 28 day | No difference in risk of death at 28 days (38.9% vs 38.7%, *P* = .94) |
|  | Interleukin-1 receptor antagonist | Fisher, 1994[144] | Original | Mortality – 28 day | No difference in risk of death at 28 days (low dose 31% vs high dose 29% vs placebo 34%, *P* = .22) |
|  |  | Opal, 1997[145] | Reproduction attempt | Mortality – 28 day | No difference in risk of death at 28 days (33.1% vs 36.4%, *P* = .36) |
|  | Tumour necrosis factor-α antibody | Abraham, 1995[146] | Original | Mortality – 28 day | No difference in risk of death at 28 days (low dose 29.5% vs high dose 31.3% vs placebo 33.1%, *P* = .33) |
|  |  | Abraham, 1998[147] | Reproduction attempt | Mortality – 28 day | No difference in risk of death at 28 days (40.3% vs 42.8%, *P* = .27) |
|  |  |  |  |  |  |
|  |  |  |  |  |  |
| **Condition** | **Clinical Practice** | **Study** | **Type of Study** | **Primary Outcome** | **Effect of Clinical Practice** |
|  | Naloxone | DeMaria, 1985[148] | Original | Increase in systolic blood pressure | No difference in blood pressure improvement (13.3% vs 11.3%, *P* =.10) |
|  |  | Safani, 1989[149] | Reproduction attempt | Not reported | No difference in survival (46% vs 54%, *P* = NS) |
| ***Nosocomial complications*** |  |  |  |  |  |
| Catheter-related bloodstream infection | Early versus late central venous catheter change | Cobb, 1992 (new insertion site)[150] | Original | Catheter-related complications | No difference in catheter-related bloodstream infection but increased mechanical complication (5% vs 1%, *P* = .005) |
|  |  | Cobb, 1992 (guidewire exchange)[150] | Original | Catheter-related complications | Non-significant trend to increased bloodstream infection with guidewire exchange (6% vs 0%, *P* = .06) |
|  |  | Chen, 2003[151] | Reproduction attempt | Catheter-related infection | No difference in catheter-related infection (OR 1.54, 95% CI 0.50-4.85) |
| **Neurological** |  |  |  |  |  |
| Traumatic brain injury | Erythropoietin | Robertson, 2014[95] | Original | Favourable outcome, Glasgow outcome scale – 6 months | No difference in likelihood of good neurological recovery  Regimen 1: OR 1.56 (95% CI 0.60-4.08)  Regimen 2: OR 1.78 (95% CI 0.66-4.83) |
|  |  | Nichol, 2015[152] | Reproduction attempt | Proportion with GOS-E score 1-4 | No difference in likelihood of good neurological recovery (RR 0.99, 95% CI 0.83 to 1.18) |
| ***Acute kidney injury*** | Continuous versus intermittent renal replacement therapy^a^ | Misset, 1996[153] | Original | None reported | No difference in mean arterial pressure |
|  |  | John, 2001[154] | Reproduction attempt | None reported | No difference in splanchnic perfusion |
|  |  | Mehta, 2001[155] | Reproduction attempt | Mortality | No difference in risk of death (OR 1.3, 95% CI 0.6-2.7) |
|  |  | Augustine, 2004[156] | Reproduction attempt | Mortality – hospital | No difference in risk of death in hospital (67.5% vs 70%, *P* = NS) |
|  |  | Uehlinger, 2005[157] | Reproduction attempt | Mortality – ICU | No difference in risk of death in ICU (34% vs 38%, *P* = .71) |
|  |  | Vinsonneau, 2006[158] | Reproduction attempt | Mortality – 60 day | No difference in risk of death at 60 days (32% vs 33%, P = .98) |
|  | N-acetylcysteine to prevent acute kidney injury in cardiac surgical patients | Burns, 2005[159] | Original | Post-operative renal dysfunction | No difference in risk of post-operative renal dysfunction (RR 1.03, 95% CI 0.72-1.46) |
|  |  | Sisillo, 2008[160] | Reproduction attempt | Acute kidney injury | No difference in acute kidney injury (ARR 12%, *P* = .06) |

ARR = absolute risk reduction; ARDS = acute respiratory distress syndrome; HR = hazard ratio; ICU = intensive care unit; NS = not statistically significant; OR = odds ratio; RR = risk ratio.

a. Continuous renal replacement therapy modalities: Misset 1996, continuous arteriovenous hemofiltration; John 2001, continuous venovenous hemofiltration; Mehta 2001, continuous arterio- or venovenous hemodiafiltration; Augustine 2004, continuous venovenous hemodialysis; Uehlinger 2005, continuous venovenous hemodiafiltration; Vinsonneau 2006, continuous venovenous hemodiafiltration.

**Table S4. Clinical practices with consistent estimates of harm between original studies and reproduction attempts.**

| **Condition** | **Intervention** | **Study** | **Type of Study** | **Primary Outcome** | **Effect of Clinical Practice** |
| --- | --- | --- | --- | --- | --- |
| ***General critical illness*** | Hydroxyethyl starch | Cittanova, 1996[161] | Original | Renal replacement therapy after transplant | Increase in use of renal replacement therapy among renal transplant recipients (33% vs 5%, *P* = .03) |
|  |  | Myburgh, 2012[162] | Reproduction attempt | Mortality – 90 day | No difference in 90-day mortality (RR 1.06, 95% CI 0.96-1.18), but increase in risk of requiring renal replacement therapy (RR 1.21, 95% CI 1.00-1.45) |
| ***Sepsis*** | Hydroxyethyl starch | Schortgen, 2001[163] | Original | Acute kidney injury | Increase risk of acute kidney injury (42% vs 23%, *P* = .03) |
|  |  | Brunkhorst, 2008[164] | Reproduction attempt | Mortality – 28 day | No difference in 28 day mortality (24.7% vs 26%, *P* = .74), but increase risk of acute kidney injury (34.9% vs 22.8%, *P* = .002) |
|  |  | Perner, 2012[165] | Reproduction attempt | Mortality – 90 day | Increase risk of death at 90 days (RR 1.17, 95% CI 1.01-1.36, *P* = .03) |
|  | Tight glycemic control | Brunkhorst, 2008[164] | Original | Mortality – 28 day | No difference in 28 day mortality (24.7% vs 26%, *P* = .74), but increase risk of experiencing severe hypoglycemia (17.0% vs 4.1%, *P* < .0001) |
|  |  | Annane, 2010[63] | Reproduction attempt | Mortality – hospital | No difference in hospital mortality (RR 1.07, 95% CI 0.88-1.30), but increase risk of severe hypoglycemia (increase of 0.15 episodes per patient, *P* = .003) |

ARDS = acute respiratory distress syndrome; ICU = intensive care unit; OR = odds ratio; RR = risk ratio.

Excluded from this table was the DECRA RCT of decompressive craniectomy in traumatic brain injury as although it showed that decompressive craniectomy worsened neurological outcome in this population (OR 1.84, 95% CI 1.05-3.24, *P* = .03)[166], it’s not clear that this will be a consistent finding until the results of its reproduction attempt, RESCUE-ICP are published [167].

**Table S5. Clinical practices with inconsistent effect estimates between original studies and reproduction attempts.**

| **Condition** | **Clinical Practice** | **Study** | **Type of Study** | **Primary**  **Outcome** | **Effect of Clinical Practice** |
| --- | --- | --- | --- | --- | --- |
| ***Efficacy to Harm*** | |  |  |  |  |
| **General critical illness** | Restrictive transfusion of red blood cells following cardiac surgery^a^ | Hajjar, 2010[168] | Original | Mortality – 30 day | Restrictive strategy non-inferior to liberal strategy (11% versus 10%, *p* = 0.85) |
|  |  | Murphy, 2015[169] | Reproduction attempt | Composite | No significant difference in risk of composite outcome (OR 1.11, 95% CI 0.91 – 1.34, *p* = 0.30); increase risk of death in restrictive group |
| ***ARDS*** | Enteral antioxidants | Gadek, 1999[170] | Original | Length of mechanical ventilation | Decrease in length of mechanical ventilation (11.0 vs 16.3 days, p = 0.01) |
|  |  | Rice, 2011[171] | Reproduction attempt | Ventilator-free days | Decrease in number of ventilator-free days (-3.2, 95% CI, -5.8 to -0.7 days, *P* = .02); Trend toward increase in risk of death in hospital (26.6% vs 16.3%, *P* = 0.054) |
| ***Efficacy to Lack of Efficacy*** | |  |  |  |  |
| ***ARDS*** | Systemic glucocorticoids | Meduri, 1998[172] | Original | Mortality – ICU | Decrease in risk of death in ICU (ARR 62%, *P* = .002) |
|  |  | Steinberg, 2006[173] | Reproduction attempt | Mortality – 60 day | No effect on risk of death at 60 days (ARR -0.6%, *P* = 1.0) |
| ***Mechanical ventilation (excluding ARDS)*** | Early tracheostomy | Rumbak, 2004[174] | Original | Ventilator-associated pneumonia | Decrease in incidence of ventilator-associated pneumonia (ARR 20%, *P*=.005) |
|  |  | Blot, 2008[175] | Reproduction attempt | Mortality – 28 day | No effect on risk of death at 28 days (20% vs 24%) |
|  |  | Terragni, 2010[176] | Reproduction attempt | Ventilator-associated pneumonia | No effect on risk of ventilator-associated pneumonia (14% vs 21%, *P* = .07) |
|  |  | Trouillet, 2011[177] | Reproduction attempt | Ventilator-free days | No difference in ventilator-free days (difference 2.1 days, 95% CI, -4.1 to 8.3 days, *P* = .5) |
|  |  | Young, 2013[178] | Reproduction attempt | Mortality – 30 day | No difference in risk of death at 30 days (ARR 0.7%, *P* = .9) |
| ***Sepsis*** | Protocolized resuscitation | Rivers, 2001[179] | Original | Mortality - hospital | Decrease in risk of death in hospital (RR 0.58, 95% CI, 0.38-0.87, *P*=.009) |
|  |  | ProCESS Investigators, 2014[180] | Reproduction attempt | Mortality – 60 day | No difference in risk of death at 60 days (RR 1.04, 95% CI, 0.82-1.31, *P*=.83) |
|  |  | ARISE Investigators, 2014[181] | Reproduction attempt | Mortality – 90 day | No difference in risk of death at 90 days (ARR 0.3%, 95% CI -3.6 to 4.1%, *p* = 0.90) |
|  |  | ProMISe Investigators, 2015[182] | Reproduction attempt | Mortality – 90 day | No difference in risk of death at 90 days (ARR -0.3%, 95% CI -5.4 to 4.7%, *p* = 0.90) |
|  | Recombinant human activated protein C | Bernard, 2001[183] | Original | Mortality – 28 day | Decrease in risk of death at 28 days (RRR 19.4%, 95% CI, 6.6-30.5%, *P*=.005) |
|  |  | Abraham, 2005[184] | Reproduction attempt | Mortality – 28 day | No difference in risk of death at 28 days (RR 1.08, 95% CI, 0.92-1.28, *P*=0.34) |
|  |  | Ranieri, 2012[185] | Reproduction attempt | Mortality – 28 day | No difference in risk of death at 28 days (RR 1.09, 95% CI, 0.92-1.28, *P*= 0.31) |
|  | Polymyxin B Hemoperfusion^b^ | Cruz, 2009[186] | Original | Change in mean arterial pressure – 72 hr | Increase in mean arterial pressure in polymyxin group (76 to 84mmHg versus 74 to 77mm Hg) |
|  |  |  |  |  |  |
| **Condition** | **Clinical Practice** | **Study** | **Type of Study** | **Primary**  **Outcome** | **Effect of Clinical Practice** |
|  |  | Payen, 2015[187] | Reproduction attempt | Mortality – 28 day | No difference in risk of death at 28 days (OR 1.59, 95% CI 0.86 – 2.94) |
| ***Nosocomial complications*** |  |  |  |  |  |
| Ventilator-associated pneumonia | Semi-recumbent positioning | Drakulovic, 1999[188] | Original | Clinically suspected pneumonia | Decrease in risk of pneumonia (ARR 26%, *P* = .003) |
|  |  | Van Nieuwenhoven, 2006[189] | Reproduction attempt | Ventilator-associated pneumonia | No difference in incidence of ventilator-associated pneumonia (10.7% vs 6.5%, *P* = NS) |
| Catheter-related bloodstream infection | Tunneled central venous catheterization | Timsit, 1996[190] | Original | Catheter-related sepsis | Decreased risk of catheter-related sepsis (OR 0.33, 95% CI, 0.13 – 0.83, *P* = .02) |
|  |  | Timsit, 1999[191] | Reproduction attempt | Catheter-related sepsis | No difference in time to catheter-related sepsis (RR 0.28, 95% CI, 0.03-1.92, *P* = .18) |
| Hospital acquired bloodstream infection | Daily bathing with chlorhexidine impregnated washcloth | Climo, 2013[192] | Original | Colonization with VRE and MRSA | Decrease in risk of colonization with antibiotic-resistant bacteria (5.1 vs 6.6 per 1000 patient-days, *P* = .3) |
|  |  | Noto, 2015[193] | Reproduction attempt | Composite-several nosocomial infections | No difference in composite outcome (rate difference -0.04, 95% CI -1.10 to 1.01, *P* = .95) |
| ***Neurological*** |  |  |  |  |  |
| Anoxic encephalopathy | Hypothermia | Bernard, 2002[194] | Original | Discharge home or to rehab facility | Increase in likelihood of discharged home or to a rehabilitation facility (OR 5.25, 95% CI, 1.47-18.76, *P*=.01) |
|  |  | Hypothermia after Cardiac Arrest Study Group, 2002[195] | Original | Neurologic outcome – 6 months | Increase in likelihood of a favourable neurological outcome 6 months after cardiac arrest (RR 1.40, 95% CI, 1.08-1.81, *P* = .009) |
|  |  | Nielsen, 2013[196] | Reproduction attempt | Mortality | No difference in risk of death or experiencing a good neurological outcome (HR 1.06, 95% CI, 0.89-1.28, *P* = .51) |
| Traumatic brain injury | Progesterone | Xiao, 2008[197] | Original | Glasgow Outcome Scale score – 3 months | Improved neurological outcome at 3 months (46% versus 30%, *P* = 0.03) |
|  |  | Wright, 2014[198] | Reproduction attempt | Extended Glasgow Outcome Scale – 6 months | No difference in outcome at 6 months (RR 0.95, 95% CI 0.85 – 1.06) |
|  |  | Skolnick, 2014[199] | Reproduction attempt | Extended Glasgow Outcome Scale – 6 months | No difference in outcome at 6 months (RR 0.96, 95% CI 0.77 – 1.18) |
| ***Acute kidney injury*** | High-intensity continuous renal replacement therapy | Ronco, 2000[200] | Original | Mortality – 15 days after stopping therapy | Increase in likelihood of survival within 15 days after stopping renal replacement therapy (35 ml/kg: HR 0.51, 95% CI, 0.36-0.72; 45 ml/kg: HR 0.49, 95% CI, 0.35-0.69) |
|  |  | Bellomo, 2009[201] | Reproduction attempt | Mortality – 90 day | No difference in risk of death at 90 days (OR 1.0, 95% CI, 0.81-1.23, *P*=0.99) |
|  | Fendolopam to prevent AKI following cardiac surgery | Cogliati, 2007[202] | Original | Acute kidney injury | Decrease in incidence AKI (ARR 15%, *P* = 0.02) |
|  |  | Bove, 2014[203] | Reproduction attempt | Renal replacement therapy | No difference in risk of requiring renal replacement therapy (20% versus 18%, P = 0.47) |
|  |  |  |  |  |  |
|  |  |  |  |  |  |
| **Condition** | **Clinical Practice** | **Study** | **Type of Study** | **Primary**  **Outcome** | **Effect of Clinical Practice** |
| ***Lack of Efficacy to Harm*** | |  |  |  |  |
| ***General critical illness*** | Dopamine (vs norepinephrine) for treatment of shock | Marik, 1994[204] | Original | Gastric mucosal pH | Dopamine: pH 7.24 to 7.18  Norepinephrine: pH 7.16 to 7.23  *P* < .001 for between group difference |
|  |  | De Backer, 2010[205] | Reproduction attempt | Mortality – 28 day | No difference in 28-day mortality (OR 1.17, 95% CI, 0.97-1.42), but increase in risk of arrhythmia (24.1% vs 12.4%, *P* < .001) |
| ***Mechanical ventilation (excluding ARDS)*** | Noninvasive ventilation as rescue therapy following extubation | Keenan, 2002[206] | Original | Re-intubation | No difference in risk of re-intubation (RR 1.04, 95% CI, 0.78-1.38, *P*=0.79) |
|  |  | Esteban, 2004[207] | Reproduction attempt | Mortality – ICU | Increase in risk of death in ICU (RR 1.78, 95% CI, 1.03-3.20, *P* = .048) |
| **Lack of Efficacy to Efficacy** | |  |  |  |  |
| ***ARDS*** | Extracorporeal life support^c^ | Zapol, 1979[208] | Original | Mortality | No difference in risk of death (ARR 1.2%) |
|  |  | Peek, 2009[209] | Reproduction attempt | Mortality or disability – 6 months | Decrease in risk of death or disability at 6 months (RR 0.69, 95% CI, 0.05-0.95, *P* = .03) |
| ***Mechanical ventilation (excluding ARDS)*** | Lung-protective ventilation in patients without ARDS | Stewart, 1998[210] | Original | Mortality – hospital | No difference in risk of death in hospital (RR 1.07, 95% CI, 0.72-1.57, *P* = .72) |
|  |  | Determann, 2010[211] | Reproduction attempt | Cytokine concentrations | Decrease in pulmonary cytokine production in lung protective group |
|  |  |  |  |  |  |
|  |  | Mascia, 2010[212] | Reproduction attempt | Number of donor lungs | Increase in number of lungs available for donation (difference 41%, 95% CI, 26.5-54.8%) |
|  | Dexmedetomidine vs midazolam | Riker, 2009[213] | Original | Time at target sedation | No difference in proportion of time at target sedation level (difference 2.20%, *P* = .18) |
|  |  | Jakob, 2012[214]^d^ | Reproduction attempt | Time at target sedation | No difference in proportion of time at target sedation level, but decrease in duration of mechanical ventilation (123 vs 164 hours, *P* = .03) |
|  | Systemic glucocorticoids to prevent laryngeal edema following extubation | Ho, 1996[215] | Original | Post-extubation stridor | No difference in risk of stridor following extubation (18% vs 26%) |
|  |  | Cheng, 2006[216] | Reproduction attempt | Post-extubation stridor | Decrease in risk of stridor following extubation (1 dose: 11.6% vs 4 doses: 7.1%, vs placebo: 30.2%, *P* < .05) |
|  |  | François, 2007[217] | Reproduction attempt | Laryngeal edema | Decrease in risk of laryngeal edema (ARR 19%, *P* < .0001) |
| ***Harm to Lack of Efficacy*** | |  |  |  |  |
| ***Sepsis*** | Tumour necrosis factor-α receptor fusion protein | Fisher, 1996[218] | Original | Mortality – 28 day | Increase in risk of death at 28 days associated with higher doses of study drug (48% vs 30%, *P* = .02) |
|  |  | Abraham, 1997[219] | Reproduction attempt | Mortality | No effect on the risk of death (ARR 15%, *P* = .3) |
| ***Nosocomial complications*** |  |  |  |  |  |
| Ventilator-associated pneumonia | Stress ulcer prophylaxis - histamine-2 receptor antagonists compared with sucralfate | Driks, 1987[220] | Original | Ventilator-associated pneumonia | Increase in risk of ventilator-associated pneumonia associated with histamine-2 receptor antagonists (ARR 11.7%, *P* = .11) |
| **Condition** | **Clinical Practice** | **Study** | **Type of Study** | **Primary**  **Outcome** | **Effect of Clinical Practice** |
|  |  | Cook, 1998[221] | Reproduction attempt | Ventilator-associated pneumonia | No difference in risk of ventilator-associated pneumonia (RR 1.18, 95% CI ,0.92-1.51) |
| ***Acute kidney injury*** | Cuprophane hemodialysis membrane (compared with polyacrylonitrile) | Schiffl, 1994[222] | Original | Mortality | Study stopped early because of increased risk of death associated with cuprophane membrane (62% vs 35%, P = .052) |
|  |  | Jorres, 1999[223] | Reproduction attempt | Mortality | No effect on risk of death (OR 1.07, 95% CI, 0.54-2.11, P = .87) |
| ***Multiple Effect Estimate Changes*** | |  |  |  |  |
| ***General critical illness*** | Tight glycemic control | Van den Berghe, 2001[224] | Original (surgical patients) | Mortality – ICU | Decrease in risk of death in ICU (ARR 3.4%, *P* < .04) |
|  |  | Van den Berghe, 2006[225] | Original (medical patients) | Mortality – hospital | No difference in risk of death in hospital (37.3% vs 40.0%, *P* = .33) |
|  |  | Finfer, 2009[226] | Reproduction attempt | Mortality – 90 day | Increase in risk of death at 90 days (OR 1.14, 95% CI, 1.02-1.28) |
|  |  | Kalfon, 2014[227] | Reproduction attempt | Mortality – 90 day | No effect on 90-day mortality, but increase in risk of severe hypoglycemia (13.2% vs 6.2%, *P* < .001) |
|  | Supra-normal oxygen delivery | Boyd, 1993[228] | Original | Mortality – 28 day | Decrease in risk of death at 28 days (ARR 16.5%, *P* = .015) |
|  |  | Hayes, 1994[229] | Reproduction attempt | Mortality - hospital | Increase in hospital mortality associated with higher cardiac index (54% vs 34%, *P* = .04) |
|  |  | Gattinoni, 1995[230] | Reproduction attempt | Mortality – ICU | No effect on risk of death in ICU (48.6% vs 48.4%, *P* = .64) |
|  | Erythropoietin | Corwin, 1999[231] | Original | Red blood cell transfusions | Decrease in cumulative number of red blood cell units transfused (166 vs 305, *P* < .002) |
|  |  | Corwin, 2002[232] | Reproduction attempt | Red blood cell transfusions | Decrease in risk of requiring red blood cell transfusion (OR 0.67, 95% CI 0.54 – 0.83) |
|  |  | Corwin, 2007[233] | Reproduction attempt | Red blood cell transfusions | No difference in proportion receiving red blood cell transfusion (RR 0.95, 95% CI, 0.85-1.06, *P* = .34) but increase in thrombotic events |
| ***ARDS*** | High-frequency oscillatory ventilation | Derdak, 2002[234] | Original | Mortality – 30 day | Improved oxygenation, and trend toward decrease in risk of death at 30 days (ARR 15%, *P* = .10) |
|  |  | Young, 2013[235] | Reproduction attempt | Mortality – 30 day | No difference in risk of death at 30 days (41.7% vs 41.1%, *P* = .85) |
|  |  | Ferguson, 2013[236] | Reproduction attempt | Mortality – hospital | Increase in risk of death in hospital (RR 1.33, 95% CI, 1.09-1.64, *P* = .005) |
|  | Ventilation in the prone position | Gattinoni, 2001[237] | Original | Mortality – 10 day | No difference in risk of death at 10 days (RR 0.84, 95% CI, 0.56-1.27) |
|  |  | Taccone, 2009[238] | Reproduction attempt | Mortality – 28 day | No difference in risk of death at 28 days (RR 0.97, 95% CI 0.84 – 1.13, *P* = .72) |
|  |  | Guerin, 2013[239] | Reproduction attempt | Mortality – 28 day | Decrease in risk of death at 28 days (HR 0.39, 95% CI, 0.25-0.63) |
| ***Respiratory failure (not ventilated)*** | Non-invasive ventilation in immunosuppressed patients | Antonelli, 2000[240] | Original | Intubation | Decrease in risk of requiring intubation (ARR 50%, *P* = .002) |
|  |  |  |  |  |  |
| **Condition** | **Clinical Practice** | **Study** | **Type of Study** | **Primary**  **Outcome** | **Effect of Clinical Practice** |
|  |  | Hilbert, 2001[241] | Reproduction attempt | Intubation | Decrease in risk of requiring intubation (ARR 31%, *P* = .03) |
|  |  | Lemaile, 2015[242] | Reproduction attempt | Mortality – 28 day | No difference in risk of death at 28 days (ARR 3.2%, *P* = .47) |
| ***Sepsis*** | Anti-endotoxin antibody | Lachman, 1984[243] | Original | Mortality | Decrease in risk of death (ARR 40.3%, *P* < .01) |
|  |  | Greenman, 1991[244] | Reproduction attempt | Mortality – 30 day | No difference in risk of death at 30 days (RR 1.0, 95% CI, 0.7-1.4) |
|  |  | Ziegler, 1991[245] | Reproduction attempt | Mortality – 28 day | Decrease in risk of death at 28 days (ARR 19%, *P* = .014) |
|  |  | Angus, 2000[246] | Reproduction attempt | Mortality – 14 day | No difference in risk of death at 14 days (ARR 1.6%, *P* = .67) |
|  | Systemic corticosteroids^e^ | Sprung, 1984[247]  (glucocorticoid) | Original | Hospital Mortality | No difference in risk of death in hospital (Methylprednisone 76%, dexamethasone 77%, placebo 69%) |
|  |  | Veterans Administration Systemic Sepsis Cooperative Study Group, 1987[248]  (glucocorticoid) | Reproduction attempt | Mortality – 14 day | No difference in risk of death at 14 days (21% vs 22%, *P* = .97) |
|  |  | Bone, 1987[249]  (glucocorticoid) | Reproduction attempt | Incidence of shock | No difference in incidence of shock (46% vs 37%) |
|  |  | Annane, 2002[250] | Reproduction attempt | Mortality – 28 day | Decrease in risk of death among cosyntropin nonresponders (HR 0.67, 95% CI 0.47 – 0.95, *p*=0.02) |
|  |  | Sprung, 2008[251] | Reproduction attempt | Mortality – 28 day | No difference in risk of death at 28 days (39.2% vs 36.1%, *P* = .69) |
| ***Nosocomial complications*** |  |  |  |  |  |
| Ventilator-associated pneumonia | Selective decontamination of the digestive tract^f^ | Pugin, 1991[252] | Original | Secondary pneumonia | Decreased risk of secondary pneumonia (ARR 62%, *P* < .0001) |
|  |  | Hammond, 1992[253] | Reproduction attempt | Not reported – hospital mortality reported | No difference in risk of hospital mortality (18% vs 17%) |
|  |  | Gastinne, 1992[254] | Reproduction attempt | Mortality - ICU | No difference in risk of mortality in ICU (34% vs 30%, *P* = .37) |
|  |  | Verwaest, 1997[255] | Reproduction attempt | Not reported | Decrease in infections, increase in resistance, no effect on survival |
|  |  | Sanchez Garcia, 1998[256] | Reproduction attempt | Ventilator-associated pneumonia | Decrease in risk of ventilator-associated pneumonia (ARR 17.9%, P = .0001) |
|  |  | De Jonge, 2003[257] | Reproduction attempt | Mortality – ICU | Decrease in risk of death in ICU (ARR 8%, *P* = .002) |
|  |  | De Smet, 2009[258] | Reproduction attempt | Mortality | Decrease in risk of death  SDD: OR 0.83 (95% CI 0.72 – 0.97), *P* = .02  SOD: OR 0.86 (95% CI 0.74 – 0.99), *P* = .05 |
|  |  | Oostdijk, 2014[259] | Reproduction attempt | Antibiotic-resistant Gram-negative bacteria | Decrease in incidence of resistant Gram-negative organisms (ARR 6.2%, P = .0001) |
| Gastrointestinal bleeding | Stress ulcer prophylaxis | Priebe, 1980[260] | Original | Gastrointestinal bleeding | Increase in risk of gastrointestinal bleeding associated with cimetidine (18% vs 0%) |
|  |  | Noseworthy, 1987[261] | Reproduction attempt | Gastrointestinal bleeding | No difference in gastrointestinal bleeding between ranitidine and antacids (0% vs 2%) |
| **Condition** | **Clinical Practice** | **Study** | **Type of Study** | **Primary**  **Outcome** | **Effect of Clinical Practice** |
| ***Neurological*** |  |  |  |  |  |
| Traumatic brain injury | Hypothermia^g^ | Marion, 1997[262] | Original | Neurological outcome | Increase in likelihood of experiencing a good neurological outcome (RR 0.5, 95% CI, 0.2-1.2) |
|  |  | Clifton, 2001[263] | Reproduction attempt | Neurological outcome | No difference in neurological outcome (RR 1.0, 95% CI, 0.8-1.2, *P*= 0.99) |
|  |  | Andrews, 2015[264] | Reproduction attempt | Extended Glasgow Outcome Scale Score – 6 months | Hypothermia associated with worse neurological outcome (OR 1.53, 95% CI 1.02 – 2.30, *P* = .04) |
| Malignant middle cerebral artery stroke | Decompressive craniectomy | Juttler, 2007[265] | Original | Modified Rankin Scale (MRS) ≤ 3 – 6 months | Increase in survival after 30 days, but no effect on functional outcome (MRS ≤ 3 at 6 months: 47% vs 27%, *P* = .23) |
|  |  | Hofmeijer, 2009[266] | Reproduction attempt | Modified Rankin Scale (MRS) ≤ 3 – 6 months | No difference in MRS ≤ 3 at 6 months (ARR 0%, 95% CI -21% to 21%) |
|  |  | Juttler, 2014[267] | Reproduction attempt | Modified Rankin Scale (MRS) ≤ 4 – 6 months | Increase in likelihood of surviving without severe disability (OR 2.91, 95% CI 1.06 – 7.49, *P* = .04) |

Abbreviations: ARDS = acute respiratory distress syndrome; ARR = absolute risk reduction; HR = hazard ratio; NS = not statistically significant; OR = odds ratio; RR = risk ratio; RRR = relative risk reduction; SDD = selective digestive decontamination; SOD = selective oropharyngeal decontamination.

a. Ongoing reproduction attempt [268].

b. Ongoing reproduction attempt [269].

c. Ongoing reproduction attempt [270].

d. This study also examined the effects of dexmedetomidine compared to propofol, however its reproduction attempt is ongoing [271].

e. Ongoing reproduction attempt [272].

f. Ongoing reproduction attempt [273].

g. This practice is the subject of 2 ongoing reproduction attempts [274, 275].

**Figure S1. Flow diagram showing study design including electronic search strategy, article eligibility criteria, and reproducibility classification.**

**Figure S2. The relationship between time since publication of the original study and the occurrence of a first reproduction attempt.**

**Online Appendix – MEDLINE Search Strategy (April 4, 2016)**

1. Critical Illness/ or Critical Care/ or Intensive Care/ or Intensive Care Units/ or Burn Units/ or Respiratory Care Units/
2. (ICU or SICU or critical illness or critically ill or ((intensive or critical) adj3 care)).ab,ti
3. Sepsis/ or Shock,septic/
4. (sepsis or septic* or SIRS or systemic inflammatory response syndrome or blood poisoning or pyaemia or pyemia or pyohemia).ab,ti.
5. Exp Shock/
6. (cardiogenic shock or traumatic shock or hemorrhag* or haemorrhag* or surgical shock).ab,ti
7. Exp Heart arrest/ or Exp Cardiopulmonary resuscitation/ or Anoxia/
8. (heart arrest* or sudden cardiac death or cardiac arrest* or cardiopulmonary resuscitation or cardio-pulmonary resuscitation or CPR or advanced cardiac life support or ACLS or respiratory arrest or pulseless or anox* or hypox*).ab,ti.
9. Craniocerebral Trauma/ or Brain Injuries/ or Brain Hemorrhage, Traumatic/ or Diffuse Axonal Injury/
10. (craniocerebral trauma or cranio-cerebral trauma or cranio-cerebral injur* or craniocerebral injur* or crushing skull injur* or frontal region trauma or head injur* or multiple head injuries or head trauma or occipital region trauma or occipital trauma or parietal region trauma or temporal region trauma or brain injur* or brain contusion* or TBI or brain laceration* or cortical contusion* or post-traumatic encephalopath* or traumatic encephalopath* or traumatic brain hemorrhage or diffuse axonal injur*).ab,ti.
11. Exp Positive-pressure Respiration/ or Respiration, artificial/ or High-frequency Ventilation/ or Noninvasive Ventilation/ or Ventilator weaning/ or Respiratory Insufficiency/ or Lung Injury/ or Acute Lung Injury/ or Ventilator-induced Lung Injury/ or Respiratory Distress Syndrome, Adult/
12. (positive-pressure respiration or positive end-expiratory pressure or positive-pressure ventilation or continuous positive airway pressure or intermittent positive-pressure breathing or intermittent positive-pressure ventilation or artificial respiration or mechanical ventilat* or (high-frequency adj3 ventilat*) or noninvasive ventilation or non-invasive ventilation or ventilator weaning or respirator weaning or respiratory insufficiency or respiratory failure or respiratory depression or ventilatory depression or lung injur* or pulmonary injur* or ALI or acute respiratory distress syndrome or adult respiratory distress syndrome or ARDS or shock lung).ab,ti.
13. exp Acute Kidney Injury/ or Renal Replacement Therapy/ or Renal Dialysis/ or Hemofiltration/
14. ((acute adj (kidney or renal) adj (failure or insufficien* or injur*)) or AKI or ARF or renal replacement therap* or RRT or kidney replacement therap* or renal dialysis or extracorporeal dialysis or extra-corporeal dialysis or hemodialysis or CRRT or continuous venovenous hemodiafiltration or continuous veno-venous hemodiafiltration or CVVHDF or continuous venovenous hemofiltration or CVVH or multiple organ failure or multiple organ system dysfunction or multisystem organ failure or multi-system organ failure or multi-organ failure* or multiorgan failure or life support* or MODS or MSOF).ab,ti.
15. Exp extracorporeal circulation/
16. (extracorporeal circulation or extra-corporeal circulation or extracorporeal membrane oxygenation or extra-corporeal membrane oxygenation or ECMO or extracorporeal life support or extra-corporeal life support or ECLS).ab,ti.
17. or/1-16
18. limit 17 to systematic reviews
19. randomized controlled trial.pt.
20. controlled clinical trial.pt.
21. randomized.ab.
22. placebo.ab.
23. clinical trials as topic.sh.
24. randomly.ab.
25. trial.ti.
26. or/19-25
27. exp Animals/ not (exp Animals/ and Humans/)
28. 26 not 27
29. 17 and 28
30. **18 or 29**
31. exp Animals/ not (exp Animals/ and Humans/)
32. 30 not 31
33. (comment or editorial or interview or letter or news).pt.
34. 32 not 33
35. New England Journal of Medicine.jn.
36. lancet.jn.
37. (JAMA or Journal of the American Medical Association).jn.
38. or/35-37
39. 34 and 38

Online-only REFERENCES

1. Hebert PC, Wells G, Blajchman MA, Marshall J, Martin C, Pagliarello G et al. A multicenter, randomized, controlled clinical trial of transfusion requirements in critical care. Transfusion Requirements in Critical Care Investigators, Canadian Critical Care Trials Group.[Erratum appears in N Engl J Med 1999 Apr 1;340(13):1056]. N Engl J Med. 1999;340:409-417.

2. Heidegger CP, Berger MM, Graf S, Zingg W, Darmon P, Costanza MC et al. Optimisation of energy provision with supplemental parenteral nutrition in critically ill patients: a randomised controlled clinical trial. Lancet. 2013;381:385-393.

3. Feeley TW, Saumarez R, Klick JM, McNabb TG, Skillman JJ. Positive end-expiratory pressure in weaning patients from controlled ventilation. A prospective randomised trial. Lancet. 1975;2:725-729.

4. Schweickert WD, Pohlman MC, Pohlman AS, Nigos C, Pawlik AJ, Esbrook CL et al. Early physical and occupational therapy in mechanically ventilated, critically ill patients: a randomised controlled trial. Lancet. 2009;373:1874-1882.

5. Pandharipande PP, Pun BT, Herr DL, Maze M, Girard TD, Miller RR et al. Effect of sedation with dexmedetomidine vs lorazepam on acute brain dysfunction in mechanically ventilated patients: the MENDS randomized controlled trial. JAMA. 2007;298:2644-2653.

6. Strom T, Martinussen T, Toft P. A protocol of no sedation for critically ill patients receiving mechanical ventilation: a randomised trial. Lancet. 2010;375:475-480.

7. Chlan LL, Weinert CR, Heiderscheit A, Tracy MF, Skaar DJ, Guttormson JL et al. Effects of patient-directed music intervention on anxiety and sedative exposure in critically ill patients receiving mechanical ventilatory support: a randomized clinical trial. JAMA. 2013;309:2335-2344.

8. Jubran A, Grant BJ, Duffner LA, Collins EG, Lanuza DM, Hoffman LA et al. Effect of pressure support vs unassisted breathing through a tracheostomy collar on weaning duration in patients requiring prolonged mechanical ventilation: a randomized trial. JAMA. 2013;309:671-677.

9. Nouira S, Marghli S, Belghith M, Besbes L, Elatrous S, Abroug F. Once daily oral ofloxacin in chronic obstructive pulmonary disease exacerbation requiring mechanical ventilation: a randomised placebo-controlled trial. Lancet. 2001;358:2020-2025.

10. Torres A, Sibila O, Ferrer M, Polverino E, Menendez R, Mensa J et al. Effect of corticosteroids on treatment failure among hospitalized patients with severe community-acquired pneumonia and high inflammatory response: a randomized clinical trial. JAMA. 2015;313:677-686.

11. Stephan F, Barrucand B, Petit P, Rezaiguia-Delclaux S, Medard A, Delannoy B et al. High-Flow Nasal Oxygen vs Noninvasive Positive Airway Pressure in Hypoxemic Patients After Cardiothoracic Surgery: A Randomized Clinical Trial. JAMA. 2015;313:2331-2339.

12. Hernandez G, Vaquero C, Gonzalez P, Subira C, Frutos-Vivar F, Rialp G et al. Effect of Postextubation High-Flow Nasal Cannula vs Conventional Oxygen Therapy on Reintubation in Low-Risk Patients: A Randomized Clinical Trial. JAMA. 2016;315:1354-1361.

13. Roberts DE, Dobson KE, Hall KW, Light RB. Effects of prolonged naloxone infusion in septic shock. Lancet. 1988;2:699-702.

14. Guesde R, Barrou B, Leblanc I, Ourahma S, Goarin JP, Coriat P et al. Administration of desmopressin in brain-dead donors and renal function in kidney recipients. Lancet. 1998;352:1178-1181.

15. Niemann CU, Feiner J, Swain S, Bunting S, Friedman M, Crutchfield M et al. Therapeutic Hypothermia in Deceased Organ Donors and Kidney-Graft Function. N Engl J Med. 2015;373:405-414.

16. Temkin NR, Dikmen SS, Wilensky AJ, Keihm J, Chabal S, Winn HR. A randomized, double-blind study of phenytoin for the prevention of post-traumatic seizures. N Engl J Med. 1990;323:497-502.

17. Chastre J, Wolff M, Fagon JY, Chevret S, Thomas F, Wermert D et al. Comparison of 8 vs 15 days of antibiotic therapy for ventilator-associated pneumonia in adults: a randomized trial. JAMA. 2003;290:2588-2598.

18. Kollef MH, Afessa B, Anzueto A, Veremakis C, Kerr KM, Margolis BD et al. Silver-coated endotracheal tubes and incidence of ventilator-associated pneumonia: the NASCENT randomized trial. JAMA. 2008;300:805-813.

19. Flowers RH, 3rd, Schwenzer KJ, Kopel RF, Fisch MJ, Tucker SI, Farr BM. Efficacy of an attachable subcutaneous cuff for the prevention of intravascular catheter-related infection. A randomized, controlled trial. JAMA. 1989;261:878-883.

20. Kamal GD, Pfaller MA, Rempe LE, Jebson PJ. Reduced intravascular catheter infection by antibiotic bonding. A prospective, randomized, controlled trial. JAMA. 1991;265:2364-2368.

21. Maki DG, Ringer M, Alvarado CJ. Prospective randomised trial of povidone-iodine, alcohol, and chlorhexidine for prevention of infection associated with central venous and arterial catheters. Lancet. 1991;338:339-343.

22. Timsit JF, Schwebel C, Bouadma L, Geffroy A, Garrouste-Orgeas M, Pease S et al. Chlorhexidine-impregnated sponges and less frequent dressing changes for prevention of catheter-related infections in critically ill adults: a randomized controlled trial. JAMA. 2009;301:1231-1241.

23. Huang SS, Septimus E, Kleinman K, Moody J, Hickok J, Avery TR et al. Targeted versus universal decolonization to prevent ICU infection.[Erratum appears in N Engl J Med. 2013 Aug 8;369(6):587]. N Engl J Med. 2013;368:2255-2265.

24. Baumgartner JD, Glauser MP, McCutchan JA, Ziegler EJ, van Melle G, Klauber MR et al. Prevention of gram-negative shock and death in surgical patients by antibody to endotoxin core glycolipid. Lancet. 1985;2:59-63.

25. The Intravenous Immunoglobulin Collaborative Study Group. Prophylactic intravenous administration of standard immune globulin as compared with core-lipopolysaccharide immune globulin in patients at high risk of postsurgical infection. N Engl J Med. 1992;327:234-240.

26. Hastings PR, Skillman JJ, Bushnell LS, Silen W. Antacid titration in the prevention of acute gastrointestinal bleeding: a controlled, randomized trial in 100 critically ill patients. N Engl J Med. 1978;298:1041-1045.

27. Inman KJ, Sibbald WJ, Rutledge FS, Clark BJ. Clinical utility and cost-effectiveness of an air suspension bed in the prevention of pressure ulcers. JAMA. 1993;269:1139-1143.

28. Schiffl H, Lang SM, Fischer R. Daily hemodialysis and the outcome of acute renal failure. N Engl J Med. 2002;346:305-310.

29. Sack JB, Kesselbrenner MB, Bregman D. Survival from in-hospital cardiac arrest with interposed abdominal counterpulsation during cardiopulmonary resuscitation. JAMA. 1992;267:379-385.

30. Cohen TJ, Goldner BG, Maccaro PC, Ardito AP, Trazzera S, Cohen MB et al. A comparison of active compression-decompression cardiopulmonary resuscitation with standard cardiopulmonary resuscitation for cardiac arrests occurring in the hospital. N Engl J Med. 1993;329:1918-1921.

31. Houdijk AP, Rijnsburger ER, Jansen J, Wesdorp RI, Weiss JK, McCamish MA et al. Randomised trial of glutamine-enriched enteral nutrition on infectious morbidity in patients with multiple trauma. Lancet. 1998;352:772-776.

32. Roquilly A, Mahe PJ, Seguin P, Guitton C, Floch H, Tellier AC et al. Hydrocortisone therapy for patients with multiple trauma: the randomized controlled HYPOLYTE study. JAMA. 2011;305:1201-1209.

33. Pond SM, Olson KR, Osterloh JD, Tong TG. Randomized study of the treatment of phenobarbital overdose with repeated doses of activated charcoal. JAMA. 1984;251:3104-3108.

34. White NJ, Dance DA, Chaowagul W, Wattanagoon Y, Wuthiekanun V, Pitakwatchara N. Halving of mortality of severe melioidosis by ceftazidime. Lancet. 1989;2:697-701.

35. Sainio V, Kemppainen E, Puolakkainen P, Taavitsainen M, Kivisaari L, Valtonen V et al. Early antibiotic treatment in acute necrotising pancreatitis. Lancet. 1995;346:663-667.

36. Stacpoole PW, Wright EC, Baumgartner TG, Bersin RM, Buchalter S, Curry SH et al. A controlled clinical trial of dichloroacetate for treatment of lactic acidosis in adults. The Dichloroacetate-Lactic Acidosis Study Group. N Engl J Med. 1992;327:1564-1569.

37. Finfer S, Bellomo R, Boyce N, French J, Myburgh J, Norton R et al. A comparison of albumin and saline for fluid resuscitation in the intensive care unit. N Engl J Med. 2004;350:2247-2256.

38. Annane D, Siami S, Jaber S, Martin C, Elatrous S, Declere AD et al. Effects of fluid resuscitation with colloids vs crystalloids on mortality in critically ill patients presenting with hypovolemic shock: the CRISTAL randomized trial. JAMA. 2013;310:1809-1817.

39. Doig GS, Simpson F, Sweetman EA, Finfer SR, Cooper DJ, Heighes PT et al. Early parenteral nutrition in critically ill patients with short-term relative contraindications to early enteral nutrition: a randomized controlled trial. JAMA. 2013;309:2130-2138.

40. Harvey SE, Parrott F, Harrison DA, Bear DE, Segaran E, Beale R et al. Trial of the route of early nutritional support in critically ill adults. N Engl J Med. 2014;371:1673-1684.

41. Amrein K, Schnedl C, Holl A, Riedl R, Christopher KB, Pachler C et al. Effect of high-dose vitamin D3 on hospital length of stay in critically ill patients with vitamin D deficiency: the VITdAL-ICU randomized clinical trial.[Erratum appears in JAMA. 2014 Nov 12;312(18):1932]. JAMA. 2014;312:1520-1530.

42. Young P, Bailey M, Beasley R, Henderson S, Mackle D, McArthur C et al. Effect of a Buffered Crystalloid Solution vs Saline on Acute Kidney Injury Among Patients in the Intensive Care Unit: The SPLIT Randomized Clinical Trial.[Erratum appears in JAMA. 2015 Dec 15;314(23):2570; PMID: 26670980]. JAMA. 2015;314:1701-1710.

43. Lacroix J, Hebert PC, Fergusson DA, Tinmouth A, Cook DJ, Marshall JC et al. Age of transfused blood in critically ill adults. N Engl J Med. 2015;372:1410-1418.

44. Bernard GR, Luce JM, Sprung CL, Rinaldo JE, Tate RM, Sibbald WJ et al. High-dose corticosteroids in patients with the adult respiratory distress syndrome. N Engl J Med. 1987;317:1565-1570.

45. Anzueto A, Baughman RP, Guntupalli KK, Weg JG, Wiedemann HP, Raventos AA et al. Aerosolized surfactant in adults with sepsis-induced acute respiratory distress syndrome. Exosurf Acute Respiratory Distress Syndrome Sepsis Study Group. N Engl J Med. 1996;334:1417-1421.

46. Ranieri VM, Suter PM, Tortorella C, De Tullio R, Dayer JM, Brienza A et al. Effect of mechanical ventilation on inflammatory mediators in patients with acute respiratory distress syndrome: a randomized controlled trial. JAMA. 1999;282:54-61.

47. ARDS Clinical Trials Network. Ketoconazole for early treatment of acute lung injury and acute respiratory distress syndrome: a randomized controlled trial. The ARDS Network.[Erratum appears in JAMA 2000 Nov 15;284(19):2450], [Erratum appears in JAMA 2001 Oct 3;286(13):1578], [Erratum appears in JAMA 2200 Nov 22-29;284(20):2597]. JAMA. 2000;283:1995-2002.

48. ARDS Clinical Trials Network. Wiedemann HP, Wheeler AP, Bernard GR, Thompson BT et al. Comparison of two fluid-management strategies in acute lung injury. N Engl J Med. 2006;354:2564-2575.

49. ARDS Clinical Trials Network. Rice TW, Wheeler AP, Thompson BT, Steingrub J et al. Initial trophic vs full enteral feeding in patients with acute lung injury: the EDEN randomized trial. JAMA. 2012;307:795-803.

50. Pepe PE, Hudson LD, Carrico CJ. Early application of positive end-expiratory pressure in patients at risk for the adult respiratory-distress syndrome. N Engl J Med. 1984;311:281-286.

51. Mehta S, Burry L, Cook D, Fergusson D, Steinberg M, Granton J et al. Daily sedation interruption in mechanically ventilated critically ill patients cared for with a sedation protocol: a randomized controlled trial.[Erratum appears in JAMA. 2013 Jan 16;309(3):237]. JAMA. 2012;308:1985-1992.

52. Guerin C, Gaillard S, Lemasson S, Ayzac L, Girard R, Beuret P et al. Effects of systematic prone positioning in hypoxemic acute respiratory failure: a randomized controlled trial. JAMA. 2004;292:2379-2387.

53. Heyland D, Muscedere J, Wischmeyer PE, Cook D, Jones G, Albert M et al. A randomized trial of glutamine and antioxidants in critically ill patients.[Erratum appears in N Engl J Med. 2013 May 9;368(19):1853 Note: Dosage error in article text.]. N Engl J Med. 2013;368:1489-1497.

54. van Zanten AR, Sztark F, Kaisers UX, Zielmann S, Felbinger TW, Sablotzki AR et al. High-protein enteral nutrition enriched with immune-modulating nutrients vs standard high-protein enteral nutrition and nosocomial infections in the ICU: a randomized clinical trial. JAMA. 2014;312:514-524.

55. Faisy C, Meziani F, Planquette B, Clavel M, Gacouin A, Bornstain C et al. Effect of Acetazolamide vs Placebo on Duration of Invasive Mechanical Ventilation Among Patients With Chronic Obstructive Pulmonary Disease: A Randomized Clinical Trial. JAMA. 2016;315:480-488.

56. Edwards G, Leszczynski SO. A double-blind trial of five respiratory stimulants in patients in acute ventilatory failure. Lancet. 1967;290:226-229.

57. Delclaux C, L'Her E, Alberti C, Mancebo J, Abroug F, Conti G et al. Treatment of acute hypoxemic nonhypercapnic respiratory insufficiency with continuous positive airway pressure delivered by a face mask: A randomized controlled trial. JAMA. 2000;284:2352-2360.

58. Jabre P, Combes X, Lapostolle F, Dhaouadi M, Ricard-Hibon A, Vivien B et al. Etomidate versus ketamine for rapid sequence intubation in acutely ill patients: a multicentre randomised controlled trial. Lancet. 2009;374:293-300.

59. Frat JP, Thille AW, Mercat A, Girault C, Ragot S, Perbet S et al. High-flow oxygen through nasal cannula in acute hypoxemic respiratory failure. N Engl J Med. 2015;372:2185-2196.

60. Bernard GR, Wheeler AP, Russell JA, Schein R, Summer WR, Steinberg KP et al. The effects of ibuprofen on the physiology and survival of patients with sepsis. The Ibuprofen in Sepsis Study Group. N Engl J Med. 1997;336:912-918.

61. Fein AM, Bernard GR, Criner GJ, Fletcher EC, Good JT, Jr., Knaus WA et al. Treatment of severe systemic inflammatory response syndrome and sepsis with a novel bradykinin antagonist, deltibant (CP-0127). Results of a randomized, double-blind, placebo-controlled trial. CP-0127 SIRS and Sepsis Study Group. JAMA. 1997;277:482-487.

62. Abraham E, Reinhart K, Opal S, Demeyer I, Doig C, Rodriguez AL et al. Efficacy and safety of tifacogin (recombinant tissue factor pathway inhibitor) in severe sepsis: a randomized controlled trial. JAMA. 2003;290:238-247.

63. Annane D, Cariou A, Maxime V, Azoulay E, D'Honneur G et al. Corticosteroid treatment and intensive insulin therapy for septic shock in adults: a randomized controlled trial.[Erratum appears in JAMA. 2010 May 5;303(17):1698]. JAMA. 2010;303:341-348.

64. Opal SM, Laterre PF, Francois B, LaRosa SP, Angus DC, Mira JP et al. Effect of eritoran, an antagonist of MD2-TLR4, on mortality in patients with severe sepsis: the ACCESS randomized trial. JAMA. 2013;309:1154-1162.

65. Brunkhorst FM, Oppert M, Marx G, Bloos F, Ludewig K, Putensen C et al. Effect of empirical treatment with moxifloxacin and meropenem vs meropenem on sepsis-related organ dysfunction in patients with severe sepsis: a randomized trial. JAMA. 2012;307:2390-2399.

66. Annane D, Vignon P, Renault A, Bollaert PE, Charpentier C, Martin C et al. Norepinephrine plus dobutamine versus epinephrine alone for management of septic shock: a randomised trial.[Erratum appears in Lancet. 2007 Sep 22;370(9592):1034]. Lancet. 2007;370:676-684.

67. Russell JA, Walley KR, Singer J, Gordon AC, Hebert PC, Cooper DJ et al. Vasopressin versus norepinephrine infusion in patients with septic shock. N Engl J Med. 2008;358:877-887.

68. Caironi P, Tognoni G, Masson S, Fumagalli R, Pesenti A, Romero M et al. Albumin replacement in patients with severe sepsis or septic shock. N Engl J Med. 2014;370:1412-1421.

69. Asfar P, Meziani F, Hamel JF, Grelon F, Megarbane B, Anguel N et al. High versus low blood-pressure target in patients with septic shock. N Engl J Med. 2014;370:1583-1593.

70. Young P, Saxena M, Bellomo R, Freebairn R, Hammond N, van Haren F et al. Acetaminophen for Fever in Critically Ill Patients with Suspected Infection. N Engl J Med. 2015;373:2215-2224.

71. Holst LB, Haase N, Wetterslev J, Wernerman J, Guttormsen AB, Karlsson S et al. Lower versus higher hemoglobin threshold for transfusion in septic shock. N Engl J Med. 2014;371:1381-1391.

72. Young B, Runge JW, Waxman KS, Harrington T, Wilberger J, Muizelaar JP et al. Effects of pegorgotein on neurologic outcome of patients with severe head injury. A multicenter, randomized controlled trial. JAMA. 1996;276:538-543.

73. Brain Resuscitation Clinical Trial I Study Group. Randomized clinical study of thiopental loading in comatose survivors of cardiac arrest. N Engl J Med. 1986;314:397-403.

74. Roine RO, Kaste M, Kinnunen A, Nikki P, Sarna S, Kajaste S. Nimodipine after resuscitation from out-of-hospital ventricular fibrillation. A placebo-controlled, double-blind, randomized trial. JAMA. 1990;264:3171-3177.

75. Roine RO, Kajaste S, Kaste M. Neuropsychological sequelae of cardiac arrest. JAMA. 1993;269:237-242.

76. Brain Resuscitation Clinical trial II Study Group. A randomized clinical study of a calcium-entry blocker (lidoflazine) in the treatment of comatose survivors of cardiac arrest. N Engl J Med. 1991;324:1225-1231.

77. Leppik IE, Derivan AT, Homan RW, Walker J, Ramsay RE, Patrick B. Double-blind study of lorazepam and diazepam in status epilepticus. JAMA. 1983;249:1452-1454.

78. Papazian L, Roch A, Charles PE, Penot-Ragon C, Perrin G, Roulier P et al. Effect of statin therapy on mortality in patients with ventilator-associated pneumonia: a randomized clinical trial. JAMA. 2013;310:1692-1700.

79. Huskins WC, Huckabee CM, O'Grady NP, Murray P, Kopetskie H, Zimmer L et al. Intervention to reduce transmission of resistant bacteria in intensive care. N Engl J Med. 2011;364:1407-1418.

80. Harris AD, Pineles L, Belton B, Johnson JK, Shardell M, Loeb M et al. Universal glove and gown use and acquisition of antibiotic-resistant bacteria in the ICU: a randomized trial. JAMA. 2013;310:1571-1580.

81. PROTECT Investigators. Cook D, Meade M, Guyatt G, Walter S, Heels-Ansdell D et al. Dalteparin versus unfractionated heparin in critically ill patients. N Engl J Med. 2011;364:1305-1314.

82. Allgren RL, Marbury TC, Rahman SN, Weisberg LS, Fenves AZ, Lafayette RA et al. Anaritide in acute tubular necrosis. Auriculin Anaritide Acute Renal Failure Study Group. N Engl J Med. 1997;336:828-834.

83. Bellomo R, Chapman M, Finfer S, Hickling K, Myburgh J. Low-dose dopamine in patients with early renal dysfunction: a placebo-controlled randomised trial. Australian and New Zealand Intensive Care Society (ANZICS) Clinical Trials Group. Lancet. 2000;356:2139-2143.

84. Phu NH, Hien TT, Mai NT, Chau TT, Chuong LV, Loc PP et al. Hemofiltration and peritoneal dialysis in infection-associated acute renal failure in Vietnam. N Engl J Med. 2002;347:895-902.

85. Palevsky PM, Zhang JH, O'Connor TZ, Chertow GM, Crowley ST et al. Intensity of renal support in critically ill patients with acute kidney injury.[Erratum appears in N Engl J Med. 2009 Dec 10;361(24):2391]. N Engl J Med. 2008;359:7-20.

86. Taylor GJ, Rubin R, Tucker M, Greene HL, Rudikoff MT, Weisfeldt ML. External cardiac compression. A randomized comparison of mechanical and manual techniques. JAMA. 1978;240:644-646.

87. Thel MC, Armstrong AL, McNulty SE, Califf RM, O'Connor CM. Randomised trial of magnesium in in-hospital cardiac arrest. Duke Internal Medicine Housestaff. Lancet. 1997;350:1272-1276.

88. Stiell IG, Hebert PC, Weitzman BN, Wells GA, Raman S, Stark RM et al. High-dose epinephrine in adult cardiac arrest. N Engl J Med. 1992;327:1045-1050.

89. Stiell IG, Hebert PC, Wells GA, Vandemheen KL, Tang AS, Higginson LA et al. Vasopressin versus epinephrine for inhospital cardiac arrest: a randomised controlled trial. Lancet. 2001;358:105-109.

90. Casaer MP, Mesotten D, Hermans G, Wouters PJ, Schetz M, Meyfroidt G et al. Early versus late parenteral nutrition in critically ill adults. N Engl J Med. 2011;365:506-517.

91. Takala J, Ruokonen E, Webster NR, Nielsen MS, Zandstra DF, Vundelinckx G et al. Increased mortality associated with growth hormone treatment in critically ill adults. N Engl J Med. 1999;341:785-792.

92. Gao Smith F, Perkins GD, Gates S, Young D, McAuley DF, Tunnicliffe W et al. Effect of intravenous beta-2 agonist treatment on clinical outcomes in acute respiratory distress syndrome (BALTI-2): a multicentre, randomised controlled trial. Lancet. 2012;379:229-235.

93. Day NP, Phu NH, Bethell DP, Mai NT, Chau TT, Hien TT et al. The effects of dopamine and adrenaline infusions on acid-base balance and systemic haemodynamics in severe infection.[Erratum appears in Lancet 1996 Sep 28;348(9031):902]. Lancet. 1996;348:219-223.

94. van Eijk MM, Roes KC, Honing ML, Kuiper MA, Karakus A, van der Jagt M et al. Effect of rivastigmine as an adjunct to usual care with haloperidol on duration of delirium and mortality in critically ill patients: a multicentre, double-blind, placebo-controlled randomised trial. Lancet. 2010;376:1829-1837.

95. Robertson CS, Hannay HJ, Yamal JM, Gopinath S, Goodman JC, Tilley BC et al. Effect of erythropoietin and transfusion threshold on neurological recovery after traumatic brain injury: a randomized clinical trial. JAMA. 2014;312:36-47.

96. Mourvillier B, Tubach F, van de Beek D, Garot D, Pichon N, Georges H et al. Induced hypothermia in severe bacterial meningitis: a randomized clinical trial. JAMA. 2013;310:2174-2183.

97. Billings FTt, Hendricks PA, Schildcrout JS, Shi Y, Petracek MR, Byrne JG et al. High-Dose Perioperative Atorvastatin and Acute Kidney Injury Following Cardiac Surgery: A Randomized Clinical Trial. JAMA. 2016;315:877-888.

98. Sloan EP, Koenigsberg M, Gens D, Cipolle M, Runge J, Mallory MN et al. Diaspirin cross-linked hemoglobin (DCLHb) in the treatment of severe traumatic hemorrhagic shock: a randomized controlled efficacy trial. JAMA. 1999;282:1857-1864.

99. Amato MB, Barbas CS, Medeiros DM, Magaldi RB, Schettino GP, Lorenzi-Filho G et al. Effect of a protective-ventilation strategy on mortality in the acute respiratory distress syndrome. N Engl J Med. 1998;338:347-354.

100. ARDS Clinical Trials Network. Ventilation with lower tidal volumes as compared with traditional tidal volumes for acute lung injury and the acute respiratory distress syndrome. N Engl J Med. 2000;342:1301-1308.

101. Villar JS, Kacmarek RM, Perez-Mendez L, Aguirre-Jaime A. A high positive end-expiratory pressure, low tidal volume ventilatory strategy improves outcome in persistent acute respiratory distress syndrome: A randomized, controlled trial*. Crit Care Med. 2006;34:1311-1318.

102. Aitkenhead AR, Pepperman ML, Willatts SM, Coates PD, Park GR, Bodenham AR et al. Comparison of propofol and midazolam for sedation in critically ill patients. Lancet. 1989;2:704-709.

103. Chamorro C, de Latorre FJ, Montero A, Sánchez-Izquierdo JA, Jareño A, Moreno JA et al. Comparative study of propofol versus midazolam in the sedation of critically ill patients: results of a prospective, randomized, multicenter trial. Crit Care Med. 1996;24:932-939.

104. Kress JP, Pohlman AS, O'Connor MF, Hall JB. Daily interruption of sedative infusions in critically ill patients undergoing mechanical ventilation. N Engl J Med. 2000;342:1471-1477.

105. Girard TD, Kress JP, Fuchs BD, Thomason JW, Schweickert WD, Pun BT et al. Efficacy and safety of a paired sedation and ventilator weaning protocol for mechanically ventilated patients in intensive care (Awakening and Breathing Controlled trial): a randomised controlled trial. Lancet. 2008;371:126-134.

106. Brochard L, Rauss A, Benito S, Conti G. Comparison of three methods of gradual withdrawal from ventilatory support during weaning from mechanical ventilation. Am J Resp Crit Care Med. 1994;150:896-903.

107. Esteban A, Frutos F, Tobin MJ, Alia I, Solsona JF, Valverdu I et al. A comparison of four methods of weaning patients from mechanical ventilation. Spanish Lung Failure Collaborative Group. N Engl J Med. 1995;332:345-350.

108. Kramer N, Meyer TJ, Meharg J, Cece RD. Randomized, prospective trial of noninvasive positive pressure ventilation in acute respiratory failure. Am J Resp Crit Care Med. 1995;151:1799-1806.

109. Antonelli M, Conti G, Rocco M, Bufi M, De Blasi RA, Vivino G et al. A comparison of noninvasive positive-pressure ventilation and conventional mechanical ventilation in patients with acute respiratory failure. N Engl J Med. 1998;339:429-435.

110. Nava S, Gregoretti C, Fanfulla F, Squadrone E, Grassi M, Carlucci A et al. Noninvasive ventilation to prevent respiratory failure after extubation in high-risk patients*. Crit Care Med. 2005;33:2465-2470.

111. Ferrer M, Valencia M, Nicolas JM, Bernadich O, Badia JR, Torres A. Early Noninvasive Ventilation Averts Extubation Failure in Patients at Risk. Am J Resp Crit Care Med. 2006;173:164-170.

112. Ferrer M, Sellares J, Valencia M, Carrillo A, Gonzalez G, Badia JR et al. Non-invasive ventilation after extubation in hypercapnic patients with chronic respiratory disorders: randomised controlled trial. Lancet. 2009;374:1082-1088.

113. Bersten AD, Holt AW, Vedig AE, Skowronski GA, Baggoley CJ. Treatment of severe cardiogenic pulmonary edema with continuous positive airway pressure delivered by face mask. N Engl J Med. 1991;325:1825-1830.

114. Masip J, Betbese AJ, Paez J, Vecilla F, Canizares R, Padro J et al. Non-invasive pressure support ventilation versus conventional oxygen therapy in acute cardiogenic pulmonary oedema: a randomised trial. Lancet. 2000;356:2126-2132.

115. Nava S, Carbone G, DiBattista N, Bellone A, Baiardi P, Cosentini R et al. Noninvasive Ventilation in Cardiogenic Pulmonary Edema. Am J Resp Crit Care Med. 2003;168:1432-1437.

116. Park M, Sangean MC, Volpe MdS, Feltrim MIZ, Nozawa E, Leite PF et al. Randomized, prospective trial of oxygen, continuous positive airway pressure, and bilevel positive airway pressure by face mask in acute cardiogenic pulmonary edema*. Crit Care Med. 2004;32:2407-2415.

117. Bott J, Carroll MP, Conway JH, Keilty SE, Ward EM, Brown AM et al. Randomised controlled trial of nasal ventilation in acute ventilatory failure due to chronic obstructive airways disease. Lancet. 1993;341:1555-1557.

118. Brochard L, Mancebo J, Wysocki M, Lofaso F, Conti G, Rauss A et al. Noninvasive ventilation for acute exacerbations of chronic obstructive pulmonary disease. N Engl J Med. 1995;333:817-822.

119. Squadrone V, Coha M, Cerutti E, Schellino MM, Biolino P, Occella P et al. Continuous positive airway pressure for treatment of postoperative hypoxemia: a randomized controlled trial. JAMA. 2005;293:589-595.

120. Jaber S, Lescot T, Futier E, Paugam-Burtz C, Seguin P, Ferrandiere M et al. Effect of Noninvasive Ventilation on Tracheal Reintubation Among Patients With Hypoxemic Respiratory Failure Following Abdominal Surgery: A Randomized Clinical Trial. JAMA. 2016;315:1345-1353.

121. Mimoz O, Pieroni L, Lawrence C, Edouard A, Costa Y, Samii K et al. Prospective, randomized trial of two antiseptic solutions for prevention of central venous or arterial catheter colonization and infection in intensive care unit patients. Crit Care Med. 1996;24:1818-1823.

122. Mimoz O, Lucet JC, Kerforne T, Pascal J, Souweine B, Goudet V et al. Skin antisepsis with chlorhexidine-alcohol versus povidone iodine-alcohol, with and without skin scrubbing, for prevention of intravascular-catheter-related infection (CLEAN): an open-label, multicentre, randomised, controlled, two-by-two factorial trial. Lancet. 2015;386:2069-2077.

123. Merrer J, De Jonghe B, Golliot F, Lefrant JY, Raffy B, Barre E et al. Complications of femoral and subclavian venous catheterization in critically ill patients: a randomized controlled trial. JAMA. 2001;286:700-707.

124. Parienti JJ, Thirion M, Megarbane B, Souweine B, Ouchikhe A, Polito A et al. Femoral vs jugular venous catheterization and risk of nosocomial events in adults requiring acute renal replacement therapy: a randomized controlled trial. JAMA. 2008;299:2413-2422.

125. Parienti JJ, Mongardon N, Megarbane B, Mira JP, Kalfon P, Gros A et al. Intravascular Complications of Central Venous Catheterization by Insertion Site. N Engl J Med. 2015;373:1220-1229.

126. Papazian L, Forel JM, Gacouin A, Penot-Ragon C, Perrin G, Loundou A et al. Neuromuscular blockers in early acute respiratory distress syndrome. N Engl J Med. 2010;363:1107-1116.

127. Reevaluation of Systemic Early Neuromuscular Blockade**.** Available at: <https://clinicaltrials.gov/ct2/show/NCT02509078>

128. Jain MK, Heyland D, Dhaliwal R, Day AG, Drover J, Keefe L et al. Dissemination of the Canadian clinical practice guidelines for nutrition support: Results of a cluster randomized controlled trial. Crit Care Med. 2006;34:2362-2369.

129. Doig GS, Simpson F, Finfer S, Delaney A, Davies AR, Mitchell I et al. Effect of evidence-based feeding guidelines on mortality of critically ill adults: a cluster randomized controlled trial. JAMA. 2008;300:2731-2741.

130. Brower RG, Lanken PN, MacIntyre N, Matthay MA, Morris A, Ancukiewicz M et al. Higher versus lower positive end-expiratory pressures in patients with the acute respiratory distress syndrome. N Engl J Med. 2004;351:327-336.

131. Mercat A, Richard JC, Vielle B, Jaber S, Osman D, Diehl JL et al. Positive end-expiratory pressure setting in adults with acute lung injury and acute respiratory distress syndrome: a randomized controlled trial. JAMA. 2008;299:646-655.

132. Meade MO, Cook DJ, Guyatt GH, Slutsky AS, Arabi YM, Cooper DJ et al. Ventilation strategy using low tidal volumes, recruitment maneuvers, and high positive end-expiratory pressure for acute lung injury and acute respiratory distress syndrome: a randomized controlled trial. JAMA. 2008;299:637-645.

133. Lundin S, Mang H, Smithies M, Stenqvist O, Frostell C. Inhalation of nitric oxide in acute lung injury: results of a European multicentre study. The European Study Group of Inhaled Nitric Oxide. Intensive Care Med. 1999;25:911-919.

134. Taylor RW, Zimmerman JL, Dellinger RP, Straube RC, Criner GJ, Davis K, Jr. et al. Low-dose inhaled nitric oxide in patients with acute lung injury: a randomized controlled trial. JAMA. 2004;291:1603-1609.

135. Spragg RG, Lewis JF, Walmrath HD, Johannigman J, Bellingan G, Laterre PF et al. Effect of recombinant surfactant protein C-based surfactant on the acute respiratory distress syndrome. N Engl J Med. 2004;351:884-892.

136. Spragg RG, Taut FJH, Lewis JF, Schenk P, Ruppert C, Dean N et al. Recombinant Surfactant Protein C–based Surfactant for Patients with Severe Direct Lung Injury. Am J Resp Crit Care Med. 2011;183:1055-1061.

137. Rice TW, Mogan S, Hays MA, Bernard GR, Jensen GL, Wheeler AP. Randomized trial of initial trophic versus full-energy enteral nutrition in mechanically ventilated patients with acute respiratory failure. Crit Care Med. 2011;39:967-974.

138. Arabi YM, Aldawood AS, Haddad SH, Al-Dorzi HM, Tamim HM, Jones G et al. Permissive Underfeeding or Standard Enteral Feeding in Critically Ill Adults.[Erratum appears in N Engl J Med. 2015 Sep 24;373(13):1281; PMID: 26398094]. N Engl J Med. 2015;372:2398-2408.

139. Craig TR, Duffy MJ, Shyamsundar M, McDowell C, O&apos;Kane CM, Elborn JS et al. A Randomized Clinical Trial of Hydroxymethylglutaryl– Coenzyme A Reductase Inhibition for Acute Lung Injury (The HARP Study). Am J Resp Crit Care Med. 2011;183:620-626.

140. ARDS Clinical Trials Network. Truwit JD, Bernard GR, Steingrub J, Matthay MA et al. Rosuvastatin for sepsis-associated acute respiratory distress syndrome. N Engl J Med. 2014;370:2191-2200.

141. McAuley DF, Laffey JG, O'Kane CM, Perkins GD, Mullan B, Trinder TJ et al. Simvastatin in the acute respiratory distress syndrome. N Engl J Med. 2014;371:1695-1703.

142. Baudo F, Caimi TM, de Cataldo F, Ravizza A, Arlati S, Casella G et al. Antithrombin III (ATIII) replacement therapy in patients with sepsis and/or postsurgical complications: a controlled double-blind, randomized, multicenter study. Intensive Care Med. 1998;24:336-342.

143. Warren BL, Eid A, Singer P, Pillay SS, Carl P, Novak I et al. High-dose antithrombin III in severe sepsis: A randomized controlled trial. JAMA. 2001;286:1869-1878.

144. Fisher CJ, Jr., Dhainaut JF, Opal SM, Pribble JP, Balk RA, Slotman GJ et al. Recombinant human interleukin 1 receptor antagonist in the treatment of patients with sepsis syndrome. Results from a randomized, double-blind, placebo-controlled trial. Phase III rhIL-1ra Sepsis Syndrome Study Group. JAMA. 1994;271:1836-1843.

145. Opal SM, Fisher CJ, Dhainaut J, Vincent JL. Confirmatory interleukin-1 receptor antagonist trial in severe sepsis: A phase III, randomized, doubleblind, placebo-controlled, multicenter trial. Crit Care Med. 1997;25:1115-1124.

146. Abraham E, Wunderink R, Silverman H, Perl TM, Nasraway S, Levy H et al. Efficacy and safety of monoclonal antibody to human tumor necrosis factor alpha in patients with sepsis syndrome. A randomized, controlled, double-blind, multicenter clinical trial. TNF-alpha MAb Sepsis Study Group. JAMA. 1995;273:934-941.

147. Abraham E, Anzueto A, Gutierrez G, Tessler S, San Pedro G, Wunderink R et al. Double-blind randomised controlled trial of monoclonal antibody to human tumour necrosis factor in treatment of septic shock. NORASEPT II Study Group. Lancet. 1998;351:929-933.

148. DeMaria A, Carven DE, Heffernan JJ, McIntosh TK, Grindlinger GA, McCabe WR. Naloxone versus placebo in treatment of septic shock. Lancet. 1985;1:1363-1365.

149. Safani M, Blair J, Ross D, Waki R, Li C, Libby G. Prospective, controlled, randomized trial of naloxone infusion in early hyperdynamic septic shock. Crit Care Med. 1989;17:1004-1009.

150. Cobb DK, High KP, Sawyer RG, Sable CA, Adams RB, Lindley DA et al. A controlled trial of scheduled replacement of central venous and pulmonary-artery catheters. N Engl J Med. 1992;327:1062-1068.

151. Chen Y-Y, Yen DH-T, Yang Y-G, Liu C-Y, Wang F-D, Chou P. Comparison between replacement at 4 days and 7 days of the infection rate for pulmonary artery catheters in an intensive care unit*. Crit Care Med. 2003;31:1353-1358.

152. Nichol A, French C, Little L, Haddad S, Presneill J, Arabi Y et al. Erythropoietin in traumatic brain injury (EPO-TBI): a double-blind randomised controlled trial. Lancet. 2015;386:2499-2506.

153. Misset B, Timsit JF, Chevret S, Renaud B. A randomized cross-over comparison of the hemodynamic response to intermittent hemodialysis and continuous hemofiltration in ICU patients with acute renal failure. Intensive Care Med. 1996;22:742-746.

154. John S, Griesbach D, Baumgärtel M, Weihprecht H, Schmieder RE, Geiger H. Effects of continuous haemofiltration vs intermittent haemodialysis on systemic haemodynamics and splanchnic regional perfusion in septic shock patients: a prospective, randomized clinical trial. Nephrol Dial Transplant. 2001;16:320-327.

155. Mehta RL, McDonald B, Gabbai FB, Pahl M, Pascual MT, Farkas A et al. A randomized clinical trial of continuous versus intermittent dialysis for acute renal failure. Kidney Int. 2001;60:1154-1163.

156. Augustine JJ, Sandy D, Seifert TH, Paganini EP. A randomized controlled trial comparing intermittent with continuous dialysis in patients with ARF. Am J Kidney Dis. 2004;44:1000-1007.

157. Uehlinger DE, Jakob SM, Ferrari P, Eichelberger M, Huynh-Do U, Marti HP et al. Comparison of continuous and intermittent renal replacement therapy for acute renal failure. Nephrol Dial Transplant. 2005;20:1630-1637.

158. Vinsonneau C, Camus C, Combes A, Costa de Beauregard MA, Klouche K, Boulain T et al. Continuous venovenous haemodiafiltration versus intermittent haemodialysis for acute renal failure in patients with multiple-organ dysfunction syndrome: a multicentre randomised trial. Lancet. 2006;368:379-385.

159. Burns KE, Chu MW, Novick RJ, Fox SA, Gallo K, Martin CM et al. Perioperative N-acetylcysteine to prevent renal dysfunction in high-risk patients undergoing cabg surgery: a randomized controlled trial. JAMA. 2005;294:342-350.

160. Sisillo E, Ceriani R, Bortone F, Juliano G, Salvi L, Veglia F et al. N-acetylcysteine for prevention of acute renal failure in patients with chronic renal insufficiency undergoing cardiac surgery: A prospective, randomized, clinical trial*. Crit Care Med. 2008;36:81-86.

161. Cittanova ML, Leblanc I, Legendre C, Mouquet C, Riou B, Coriat P. Effect of hydroxyethylstarch in brain-dead kidney donors on renal function in kidney-transplant recipients. Lancet. 1996;348:1620-1622.

162. Myburgh JA, Finfer S, Bellomo R, Billot L, Cass A, Gattas D et al. Hydroxyethyl starch or saline for fluid resuscitation in intensive care. N Engl J Med. 2012;367:1901-1911.

163. Schortgen F, Lacherade JC, Bruneel F, Cattaneo I, Hemery F, Lemaire F et al. Effects of hydroxyethylstarch and gelatin on renal function in severe sepsis: a multicentre randomised study. Lancet. 2001;357:911-916.

164. Brunkhorst FM, Engel C, Bloos F, Meier-Hellmann A, Ragaller M, Weiler N et al. Intensive insulin therapy and pentastarch resuscitation in severe sepsis. N Engl J Med. 2008;358:125-139.

165. Perner A, Haase N, Guttormsen AB, Tenhunen J, Klemenzson G, Aneman A et al. Hydroxyethyl starch 130/0.42 versus Ringer's acetate in severe sepsis.[Erratum appears in N Engl J Med. 2012 Aug 2;367(5):481]. N Engl J Med. 2012;367:124-134.

166. Cooper DJ, Rosenfeld JV, Murray L, Arabi YM, Davies AR, D'Urso P et al. Decompressive craniectomy in diffuse traumatic brain injury.[Erratum appears in N Engl J Med. 2011 Nov 24;365(21):2040]. N Engl J Med. 2011;364:1493-1502.

167. Hutchinson P: Randomised Evaluation of Surgery with Craniectomy for Uncontrollable Elevation of intracranial pressure (ICP). Available at: [http://www.rescueicp.com/](http://www.RESCUEicp.com).

168. Hajjar LA, Vincent JL, Galas FR, Nakamura RE, Silva CM, Santos MH et al. Transfusion requirements after cardiac surgery: the TRACS randomized controlled trial. JAMA. 2010;304:1559-1567.

169. Murphy GJ, Pike K, Rogers CA, Wordsworth S, Stokes EA, Angelini GD et al. Liberal or restrictive transfusion after cardiac surgery.[Erratum appears in N Engl J Med. 2015 Jun 4;372(23):2274; PMID: 26039619]. N Engl J Med. 2015;372:997-1008.

170. Gadek JE, DeMichele SJ, Karlstad MD, Pacht ER, Donahoe M, Albertson TE et al. Effect of enteral feeding with eicosapentaenoic acid, gamma-linolenic acid, and antioxidants in patients with acute respiratory distress syndrome. Enteral Nutrition in ARDS Study Group. Crit Care Med. 1999;27:1409-1420.

171. Rice TW, Wheeler AP, Thompson BT, deBoisblanc BP, Steingrub J, Rock P et al. Enteral omega-3 fatty acid, gamma-linolenic acid, and antioxidant supplementation in acute lung injury.[Erratum appears in JAMA. 2012 Feb 8;307(6):563]. JAMA. 2011;306:1574-1581.

172. Meduri GU, Headley AS, Golden E, Carson SJ, Umberger RA, Kelso T et al. Effect of prolonged methylprednisolone therapy in unresolving acute respiratory distress syndrome: a randomized controlled trial. JAMA. 1998;280:159-165.

173. Steinberg KP, Hudson LD, Goodman RB, Hough CL, Lanken PN, Hyzy R et al. Efficacy and safety of corticosteroids for persistent acute respiratory distress syndrome. N Engl J Med. 2006;354:1671-1684.

174. Rumbak MJ, Newton M, Truncale T, Schwartz SW, Adams JW, Hazard PB. A prospective, randomized, study comparing early percutaneous dilational tracheotomy to prolonged translaryngeal intubation (delayed tracheotomy) in critically ill medical patients*. Crit Care Med. 2004;32:1689-1694.

175. Blot F, Similowski T, Trouillet JL, Chardon P, Korach JM, Costa MA et al. Early tracheotomy versus prolonged endotracheal intubation in unselected severely ill ICU patients. Intensive Care Med. 2008;34:1779-1787.

176. Terragni PP, Antonelli M, Fumagalli R, Faggiano C, Berardino M, Pallavicini FB et al. Early vs late tracheotomy for prevention of pneumonia in mechanically ventilated adult ICU patients: a randomized controlled trial. JAMA. 2010;303:1483-1489.

177. Trouillet J-L, Luyt C-E, Guiguet M, Ouattara A, Vaissier E, Makri R et al. Early percutaneous tracheotomy versus prolonged intubation of mechanically ventilated patients after cardiac surgery: a randomized trial. Ann Intern Med. 2011;154:373-383.

178. Young D, Harrison DA, Cuthbertson BH, Rowan K, TracMan C. Effect of early vs late tracheostomy placement on survival in patients receiving mechanical ventilation: the TracMan randomized trial. JAMA. 2013;309:2121-2129.

179. Rivers E, Nguyen B, Havstad S, Ressler J, Muzzin A, Knoblich B et al. Early goal-directed therapy in the treatment of severe sepsis and septic shock. N Engl J Med. 2001;345:1368-1377.

180. The ProCESS Ingestigators. Yealy DM, Kellum JA, Huang DT, Barnato AE, Weissfeld LA et al. A randomized trial of protocol-based care for early septic shock. N Engl J Med. 2014;370:1683-1693.

181. Peake SL, Delaney A, Bailey M, Bellomo R, Cameron PA, Cooper DJ et al. Goal-directed resuscitation for patients with early septic shock. N Engl J Med. 2014;371:1496-1506.

182. Mouncey PR, Osborn TM, Power GS, Harrison DA, Sadique MZ, Grieve RD et al. Trial of early, goal-directed resuscitation for septic shock. N Engl J Med. 2015;372:1301-1311.

183. Bernard GR, Vincent JL, Laterre PF, LaRosa SP, Dhainaut JF, Lopez-Rodriguez A et al. Efficacy and safety of recombinant human activated protein C for severe sepsis. N Engl J Med. 2001;344:699-709.

184. Abraham E, Laterre PF, Garg R, Levy H, Talwar D, Trzaskoma BL et al. Drotrecogin alfa (activated) for adults with severe sepsis and a low risk of death. N Engl J Med. 2005;353:1332-1341.

185. Ranieri VM, Thompson BT, Barie PS, Dhainaut JF, Douglas IS, Finfer S et al. Drotrecogin alfa (activated) in adults with septic shock. N Engl J Med. 2012;366:2055-2064.

186. Cruz DN, Antonelli M, Fumagalli R, Foltran F, Brienza N, Donati A et al. Early use of polymyxin B hemoperfusion in abdominal septic shock: the EUPHAS randomized controlled trial. JAMA. 2009;301:2445-2452.

187. Payen DM, Guilhot J, Launey Y, Lukaszewicz AC, Kaaki M, Veber B et al. Early use of polymyxin B hemoperfusion in patients with septic shock due to peritonitis: a multicenter randomized control trial. Intensive Care Med. 2015;41:975-984.

188. Drakulovic MB, Torres A, Bauer TT, Nicolas JM, Nogue S, Ferrer M. Supine body position as a risk factor for nosocomial pneumonia in mechanically ventilated patients: a randomised trial. Lancet. 1999;354:1851-1858.

189. van Nieuwenhoven CA, Vandenbroucke-Grauls C, van Tiel FH, Joore HCA, van Schijndel RJMS, van der Tweel I et al. Feasibility and effects of the semirecumbent position to prevent ventilator-associated pneumonia: A randomized study*. Crit Care Med. 2006;34:396-402.

190. Timsit JF, Sebille V, Farkas JC, Misset B, Martin JB, Chevret S et al. Effect of subcutaneous tunneling on internal jugular catheter-related sepsis in critically ill patients: a prospective randomized multicenter study. JAMA. 1996;276:1416-1420.

191. Timsit JF, Bruneel F, Cheval C, Mamzer MF, Garrouste-Orgeas M, Wolff M et al. Use of tunneled femoral catheters to prevent catheter-related infection. A randomized, controlled trial. Ann Intern Med. 1999;130:729-735.

192. Climo MW, Yokoe DS, Warren DK, Perl TM, Bolon M, Herwaldt LA et al. Effect of daily chlorhexidine bathing on hospital-acquired infection.[Erratum appears in N Engl J Med. 2013 Jun 13;368(24):2341]. N Engl J Med. 2013;368:533-542.

193. Noto MJ, Domenico HJ, Byrne DW, Talbot T, Rice TW, Bernard GR et al. Chlorhexidine bathing and health care-associated infections: a randomized clinical trial. JAMA. 2015;313:369-378.

194. Bernard SA, Gray TW, Buist MD, Jones BM, Silvester W, Gutteridge G et al. Treatment of comatose survivors of out-of-hospital cardiac arrest with induced hypothermia. N Engl J Med. 2002;346:557-563.

195. Hypothermia after Cardiac Arrest Study Group. Mild therapeutic hypothermia to improve the neurologic outcome after cardiac arrest.[Erratum appears in N Engl J Med 2002 May 30;346(22):1756]. N Engl J Med. 2002;346:549-556.

196. Nielsen N, Wetterslev J, Cronberg T, Erlinge D, Gasche Y, Hassager C et al. Targeted temperature management at 33degreeC versus 36degreeC after cardiac arrest. N Engl J Med. 2013;369:2197-2206.

197. Xiao G, Wei J, Yan W, Wang W, Lu Z. Improved outcomes from the administration of progesterone for patients with acute severe traumatic brain injury: a randomized controlled trial. Crit Care. 2008;12:R61.

198. Wright DW, Yeatts SD, Silbergleit R, Palesch YY, Hertzberg VS, Frankel M et al. Very early administration of progesterone for acute traumatic brain injury. N Engl J Med. 2014;371:2457-2466.

199. Skolnick BE, Maas AI, Narayan RK, van der Hoop RG, MacAllister T, Ward JD et al. A clinical trial of progesterone for severe traumatic brain injury. N Engl J Med. 2014;371:2467-2476.

200. Ronco C, Bellomo R, Homel P, Brendolan A, Dan M, Piccinni P et al. Effects of different doses in continuous veno-venous haemofiltration on outcomes of acute renal failure: a prospective randomised trial. Lancet. 2000;356:26-30.

201. Bellomo R, Cass A, Cole L, Finfer S, Gallagher M, Lo S et al. Intensity of continuous renal-replacement therapy in critically ill patients. N Engl J Med. 2009;361:1627-1638.

202. Cogliati AA, Vellutini R, Nardini A, Urovi S, Hamdan M, Landoni G et al. Fenoldopam infusion for renal protection in high-risk cardiac surgery patients: a randomized clinical study. J Cardiothorac Vasc Anesth. 2007;21:847-850.

203. Bove T, Zangrillo A, Guarracino F, Alvaro G, Persi B, Maglioni E et al. Effect of fenoldopam on use of renal replacement therapy among patients with acute kidney injury after cardiac surgery: a randomized clinical trial. JAMA. 2014;312:2244-2253.

204. Marik PE, Mohedin M. The contrasting effects of dopamine and norepinephrine on systemic and splanchnic oxygen utilization in hyperdynamic sepsis. JAMA. 1994;272:1354-1357.

205. De Backer D, Biston P, Devriendt J, Madl C, Chochrad D, Aldecoa C et al. Comparison of dopamine and norepinephrine in the treatment of shock. N Engl J Med. 2010;362:779-789.

206. Keenan SP, Powers C, McCormack DG, Block G. Noninvasive positive-pressure ventilation for postextubation respiratory distress: a randomized controlled trial. JAMA. 2002;287:3238-3244.

207. Esteban A, Frutos-Vivar F, Ferguson ND, Arabi Y, Apezteguia C, Gonzalez M et al. Noninvasive positive-pressure ventilation for respiratory failure after extubation. N Engl J Med. 2004;350:2452-2460.

208. Zapol WM, Snider MT, Hill JD, Fallat RJ, Bartlett RH, Edmunds LH et al. Extracorporeal membrane oxygenation in severe acute respiratory failure. A randomized prospective study. JAMA. 1979;242:2193-2196.

209. Peek GJ, Mugford M, Tiruvoipati R, Wilson A, Allen E, Thalanany MM et al. Efficacy and economic assessment of conventional ventilatory support versus extracorporeal membrane oxygenation for severe adult respiratory failure (CESAR): a multicentre randomised controlled trial.[Erratum appears in Lancet. 2009 Oct 17;374(9698):1330]. Lancet. 2009;374:1351-1363.

210. Stewart TE, Meade MO, Cook DJ, Granton JT, Hodder RV, Lapinsky SE et al. Evaluation of a ventilation strategy to prevent barotrauma in patients at high risk for acute respiratory distress syndrome. Pressure- and Volume-Limited Ventilation Strategy Group. N Engl J Med. 1998;338:355-361.

211. Determann RM, Royakkers A, Wolthuis EK, Vlaar AP, Choi G, Paulus F et al. Ventilation with lower tidal volumes as compared with conventional tidal volumes for patients without acute lung injury: a preventive randomized controlled trial. Crit Care. 2010;14:R1.

212. Mascia L, Pasero D, Slutsky AS, Arguis MJ, Berardino M, Grasso S et al. Effect of a lung protective strategy for organ donors on eligibility and availability of lungs for transplantation: a randomized controlled trial. JAMA. 2010;304:2620-2627.

213. Riker RR, Shehabi Y, Bokesch PM, Ceraso D, Wisemandle W, Koura F et al. Dexmedetomidine vs midazolam for sedation of critically ill patients: a randomized trial. JAMA. 2009;301:489-499.

214. Jakob SM, Ruokonen E, Grounds RM, Sarapohja T, Garratt C, Pocock SJ et al. Dexmedetomidine vs midazolam or propofol for sedation during prolonged mechanical ventilation: two randomized controlled trials. JAMA. 2012;307:1151-1160.

215. Ho LI, Harn HJ, Lien TC, Hu PY, Wang JH. Postextubation laryngeal edema in adults risk factor evaluation and prevention by hydrocortisone. Intensive Care Med. 1996;22:933-936.

216. Cheng K-C, Hou C-C, Huang H-C, Lin S-C, Zhang H. Intravenous injection of methylprednisolone reduces the incidence of postextubation stridor in intensive care unit patients*. Crit Care Med. 2006;34:1345-1350.

217. Francois B, Bellissant E, Gissot V, Desachy A, Normand S, Boulain T et al. 12-h pretreatment with methylprednisolone versus placebo for prevention of postextubation laryngeal oedema: a randomised double-blind trial. Lancet. 2007;369:1083-1089.

218. Fisher CJ, Jr., Agosti JM, Opal SM, Lowry SF, Balk RA, Sadoff JC et al. Treatment of septic shock with the tumor necrosis factor receptor:Fc fusion protein. The Soluble TNF Receptor Sepsis Study Group. N Engl J Med. 1996;334:1697-1702.

219. Abraham E, Glauser MP, Butler T, Garbino J, Gelmont D, Laterre PF et al. p55 Tumor necrosis factor receptor fusion protein in the treatment of patients with severe sepsis and septic shock. A randomized controlled multicenter trial. Ro 45-2081 Study Group. JAMA. 1997;277:1531-1538.

220. Driks MR, Craven DE, Celli BR, Manning M, Burke RA, Garvin GM et al. Nosocomial pneumonia in intubated patients given sucralfate as compared with antacids or histamine type 2 blockers. The role of gastric colonization. N Engl J Med. 1987;317:1376-1382.

221. Cook D, Guyatt G, Marshall J, Leasa D, Fuller H, Hall R et al. A comparison of sucralfate and ranitidine for the prevention of upper gastrointestinal bleeding in patients requiring mechanical ventilation. Canadian Critical Care Trials Group. N Engl J Med. 1998;338:791-797.

222. Schiffl H, Lang SM, Konig A, Strasser T, Haider MC, Held E. Biocompatible membranes in acute renal failure: prospective case-controlled study. Lancet. 1994;344:570-572.

223. Jorres A, Gahl GM, Dobis C, Polenakovic MH, Cakalaroski K, Rutkowski B et al. Haemodialysis-membrane biocompatibility and mortality of patients with dialysis-dependent acute renal failure: A prospective randomised multicentre trial. Lancet. 1999;354:1337-1341.

224. van den Berghe G, Wouters P, Weekers F, Verwaest C, Bruyninckx F, Schetz M et al. Intensive insulin therapy in critically ill patients. N Engl J Med. 2001;345:1359-1367.

225. Van den Berghe G, Wilmer A, Hermans G, Meersseman W, Wouters PJ, Milants I et al. Intensive insulin therapy in the medical ICU. N Engl J Med. 2006;354:449-461.

226. Finfer S, Chittock DR, Su SY, Blair D, Foster D, Dhingra V et al. Intensive versus conventional glucose control in critically ill patients. N Engl J Med. 2009;360:1283-1297.

227. Kalfon P, Giraudeau B, Ichai C, Guerrini A, Brechot N, Cinotti R et al. Tight computerized versus conventional glucose control in the ICU: a randomized controlled trial. Intensive Care Med. 2014;40:171-181.

228. Boyd O, Grounds RM, Bennett ED. A randomized clinical trial of the effect of deliberate perioperative increase of oxygen delivery on mortality in high-risk surgical patients. JAMA. 1993;270:2699-2707.

229. Hayes MA, Timmins AC, Yau EH, Palazzo M, Hinds CJ, Watson D. Elevation of systemic oxygen delivery in the treatment of critically ill patients. N Engl J Med. 1994;330:1717-1722.

230. Gattinoni L, Brazzi L, Pelosi P, Latini R, Tognoni G, Pesenti A et al. A trial of goal-oriented hemodynamic therapy in critically ill patients. SvO2 Collaborative Group. N Engl J Med. 1995;333:1025-1032.

231. Corwin HL, Gettinger A, Rodriguez RM, Pearl RG, Gubler KD, Enny C et al. Efficacy of recombinant human erythropoietin in the critically ill patient: a randomized, double-blind, placebo-controlled trial. Crit Care Med. 1999;27:2346-2350.

232. Corwin HL, Gettinger A, Pearl RG, Fink MP, Levy MM, Shapiro MJ et al. Efficacy of recombinant human erythropoietin in critically ill patients: a randomized controlled trial. JAMA. 2002;288:2827-2835.

233. Corwin HL, Gettinger A, Fabian TC, May A, Pearl RG, Heard S et al. Efficacy and safety of epoetin alfa in critically ill patients. N Engl J Med. 2007;357:965-976.

234. Derdak S, Mehta S, Stewart TE, Smith T, Rogers M, Buchman TG et al. High-Frequency Oscillatory Ventilation for Acute Respiratory Distress Syndrome in Adults. Am J Resp Crit Care Med. 2002;166:801-808.

235. Young D, Lamb SE, Shah S, MacKenzie I, Tunnicliffe W, Lall R et al. High-frequency oscillation for acute respiratory distress syndrome. N Engl J Med. 2013;368:806-813.

236. Ferguson ND, Cook DJ, Guyatt GH, Mehta S, Hand L, Austin P et al. High-frequency oscillation in early acute respiratory distress syndrome. N Engl J Med. 2013;368:795-805.

237. Gattinoni L, Tognoni G, Pesenti A, Taccone P, Mascheroni D, Labarta V et al. Effect of prone positioning on the survival of patients with acute respiratory failure. N Engl J Med. 2001;345:568-573.

238. Taccone P, Pesenti A, Latini R, Polli F, Vagginelli F, Mietto C et al. Prone positioning in patients with moderate and severe acute respiratory distress syndrome: a randomized controlled trial. JAMA. 2009;302:1977-1984.

239. Guerin C, Reignier J, Richard JC, Beuret P, Gacouin A, Boulain T et al. Prone positioning in severe acute respiratory distress syndrome. N Engl J Med. 2013;368:2159-2168.

240. Antonelli M, Conti G, Bufi M, Costa MG, Lappa A, Rocco M et al. Noninvasive ventilation for treatment of acute respiratory failure in patients undergoing solid organ transplantation: a randomized trial. JAMA. 2000;283:235-241.

241. Hilbert G, Gruson D, Vargas F, Valentino R, Gbikpi-Benissan G, Dupon M et al. Noninvasive ventilation in immunosuppressed patients with pulmonary infiltrates, fever, and acute respiratory failure. N Engl J Med. 2001;344:481-487.

242. Lemiale V, Mokart D, Resche-Rigon M, Pene F, Mayaux J, Faucher E et al. Effect of Noninvasive Ventilation vs Oxygen Therapy on Mortality Among Immunocompromised Patients With Acute Respiratory Failure: A Randomized Clinical Trial. JAMA. 2015;314:1711-1719.

243. Lachman E, Pitsoe SB, Gaffin SL. Anti-lipopolysaccharide immunotherapy in management of septic shock of obstetric and gynaecological origin. Lancet. 1984;1:981-983.

244. Greenman RL, Schein RM, Martin MA, Wenzel RP, MacIntyre NR, Emmanuel G et al. A controlled clinical trial of E5 murine monoclonal IgM antibody to endotoxin in the treatment of gram-negative sepsis. The XOMA Sepsis Study Group. JAMA. 1991;266:1097-1102.

245. Ziegler EJ, Fisher CJ, Jr., Sprung CL, Straube RC, Sadoff JC, Foulke GE et al. Treatment of gram-negative bacteremia and septic shock with HA-1A human monoclonal antibody against endotoxin. A randomized, double-blind, placebo-controlled trial. The HA-1A Sepsis Study Group. N Engl J Med. 1991;324:429-436.

246. Angus DC, Birmingham MC, Balk RA, Scannon PJ, Collins D, Kruse JA et al. E5 murine monoclonal antiendotoxin antibody in gram-negative sepsis: a randomized controlled trial. E5 Study Investigators. JAMA. 2000;283:1723-1730.

247. Sprung CL, Caralis PV, Marcial EH, Pierce M, Gelbard MA, Long WM et al. The effects of high-dose corticosteroids in patients with septic shock. A prospective, controlled study. N Engl J Med. 1984;311:1137-1143.

248. The Veterans Administration Systemic Sepsis Cooperative Study Group. Effect of high-dose glucocorticoid therapy on mortality in patients with clinical signs of systemic sepsis. N Engl J Med. 1987;317:659-665.

249. Bone RC, Fisher CJ, Jr., Clemmer TP, Slotman GJ, Metz CA, Balk RA. A controlled clinical trial of high-dose methylprednisolone in the treatment of severe sepsis and septic shock. N Engl J Med. 1987;317:653-658.

250. Annane D, Sebille V, Charpentier C, Bollaert PE, Francois B, Korach JM et al. Effect of treatment with low doses of hydrocortisone and fludrocortisone on mortality in patients with septic shock.[Erratum appears in JAMA. 2008 Oct 8;300(14):1652 Note: Chaumet-Riffaut, Philippe [corrected to Chaumet-Riffaud, Philippe]]. JAMA. 2002;288:862-871.

251. Sprung CL, Annane D, Keh D, Moreno R, Singer M, Freivogel K et al. Hydrocortisone therapy for patients with septic shock. N Engl J Med. 2008;358:111-124.

252. Pugin J, Auckenthaler R, Lew DP, Suter PM. Oropharyngeal decontamination decreases incidence of ventilator-associated pneumonia. A randomized, placebo-controlled, double-blind clinical trial. JAMA. 1991;265:2704-2710.

253. Hammond JM, Potgieter PD, Saunders GL, Forder AA. Double-blind study of selective decontamination of the digestive tract in intensive care. Lancet. 1992;340:5-9.

254. Gastinne H, Wolff M, Delatour F, Faurisson F, Chevret S. A controlled trial in intensive care units of selective decontamination of the digestive tract with nonabsorbable antibiotics. The French Study Group on Selective Decontamination of the Digestive Tract. N Engl J Med. 1992;326:594-599.

255. Verwaest C, Verhaegen J, Ferdinande P, Schetz M, Van den Berghe G, Verbist L et al. Randomized, controlled trial of selective digestive decontamination in 600 mechanically ventilated patients in a multidisciplinary intensive care unit. Crit Care Med. 1997;25:63-71.

256. Sanchez Garcia M, Cambronero Galache JA, Lopez Diaz J, Cerda Cerda E, Rubio Blasco J, Gomez Aguinaga MA et al. Effectiveness and cost of selective decontamination of the digestive tract in critically ill intubated patients. A randomized, double-blind, placebo-controlled, multicenter trial. Am J Respir Crit Care Med. 1998;158:908-916.

257. de Jonge E, Schultz MJ, Spanjaard L, Bossuyt PM, Vroom MB, Dankert J et al. Effects of selective decontamination of digestive tract on mortality and acquisition of resistant bacteria in intensive care: a randomised controlled trial. Lancet. 2003;362:1011-1016.

258. de Smet AM, Kluytmans JA, Cooper BS, Mascini EM, Benus RF, van der Werf TS et al. Decontamination of the digestive tract and oropharynx in ICU patients. N Engl J Med. 2009;360:20-31.

259. Oostdijk EA, Kesecioglu J, Schultz MJ, Visser CE, de Jonge E, van Essen EH et al. Effects of decontamination of the oropharynx and intestinal tract on antibiotic resistance in ICUs: a randomized clinical trial. JAMA. 2014;312:1429-1437.

260. Priebe HJ, Skillman JJ, Bushnell LS, Long PC, Silen W. Antacid versus cimetidine in preventing acute gastrointestinal bleeding. A randomized trial in 75 critically ill patients. N Engl J Med. 1980;302:426-430.

261. Noseworthy TW, Shustack A, Johnston RG, Anderson BJ, Konopad E, Grace M. A randomized clinical trial comparing ranitidine and antacids in critically ill patients. Crit Care Med. 1987;15:817-819.

262. Marion DW, Penrod LE, Kelsey SF, Obrist WD, Kochanek PM, Palmer AM et al. Treatment of traumatic brain injury with moderate hypothermia. N Engl J Med. 1997;336:540-546.

263. Clifton GL, Miller ER, Choi SC, Levin HS, McCauley S, Smith KR, Jr. et al. Lack of effect of induction of hypothermia after acute brain injury. N Engl J Med. 2001;344:556-563.

264. Andrews PJ, Sinclair HL, Rodriguez A, Harris BA, Battison CG, Rhodes JK et al. Hypothermia for Intracranial Hypertension after Traumatic Brain Injury. N Engl J Med. 2015;373:2403-2412.

265. Juttler E, Schwab S, Schmiedek P, Unterberg A, Hennerici M, Woitzik J et al. Decompressive Surgery for the Treatment of Malignant Infarction of the Middle Cerebral Artery (DESTINY): A Randomized, Controlled Trial. Stroke. 2007;38:2518-2525.

266. Hofmeijer J, Kappelle LJ, Algra A, Amelink GJ, van Gijn J, van der Worp HB et al. Surgical decompression for space-occupying cerebral infarction (the Hemicraniectomy After Middle Cerebral Artery infarction with Life-threatening Edema Trial [HAMLET]): a multicentre, open, randomised trial. Lancet Neurol. 2009;8:326-333.

267. Juttler E, Unterberg A, Woitzik J, Bosel J, Amiri H, Sakowitz OW et al. Hemicraniectomy in older patients with extensive middle-cerebral-artery stroke. N Engl J Med. 2014;370:1091-1100.

268. Transfusion requirements in cardiac surgery III (TRICS-III). Available at: <https://clinicaltrials.gov/ct2/show/NCT02042898>]

269. Safety and Efficacy of Polymyxin B Hemoperfusion (PMX) for Septic Shock. Available at: <https://clinicaltrials.gov/ct2/show/NCT01046669>

270. Extracorporeal membrane oxygenation for severe acute respiratory distress syndrome. Available at: <https://clinicaltrials.gov/ct2/show/NCT01470703?term=NCT01470703&rank=1>

271. The MENDSII Study, Maximizing the Efficacy of Sedation and Reducing Neurological Dysfunction and Mortality in Septic Patients With Acute Respiratory Failure. Available at: <https://clinicaltrials.gov/ct2/results?term=The+MENDSII+Study%2C+Maximizing+the+Efficacy+of+Sedation+and+Reducing+Neurological+Dysfunction+and+Mortality+in+Septic+Patients+With+Acute+Respiratory+Failure&Search=Search>

272. Adjunctive corticosteroid treatment in critically ill patients with septic shock (ADRENAL). Available at: <https://clinicaltrials.gov/ct2/show/NCT01448109>

273. The SuDDICU Study of Antibiotic Prophylaxis in Critical Illness. Available at: <https://clinicaltrials.gov/ct2/show/NCT02389036>

274. The Prophylactic Hypothermia Trial to Lessen Traumatic Brain Injury. Available at: <https://clinicaltrials.gov/ct2/show/NCT00987688>

275. Randomized Controlled Trial of Long-term Mild Hypothermia for Severe Traumatic Brain Injury. Available at: <https://clinicaltrials.gov/ct2/show/NCT01886222>
